# Modulation of phenolic metabolism under stress conditions in a *Lotus japonicus* mutant lacking plastidic glutamine synthetase

**DOI:** 10.3389/fpls.2015.00760

**Published:** 2015-09-25

**Authors:** Margarita García-Calderón, Teresa Pons-Ferrer, Anna Mrázova, Peter Pal'ove-Balang, Mária Vilková, Carmen M. Pérez-Delgado, José M. Vega, Adriana Eliášová, Miroslav Repčák, Antonio J. Márquez, Marco Betti

**Affiliations:** ^1^Departamento de Bioquímica Vegetal y Biología Molecular, Facultad de Química, Universidad de SevillaSeville, Spain; ^2^Faculty of Science, Institute of Biology and Ecology, P. J. Šafárik UniversityKošice, Slovakia; ^3^Faculty of Natural Sciences, Institute of Chemistry, P. J. Šafárik UniversityKošice, Slovakia; ^4^Department of Ecology, Faculty of Humanities and Natural Sciences, University of PrešovPrešov, Slovakia

**Keywords:** *Lotus japonicus*, phenolic compounds, glutamine synthetase, flavonoids, isoflavonoids

## Abstract

This paper was aimed to investigate the possible implications of the lack of plastidic glutamine synthetase (GS_2_) in phenolic metabolism during stress responses in the model legume *Lotus japonicus*. Important changes in the transcriptome were detected in a GS_2_ mutant called *Ljgln2-2*, compared to the wild type, in response to two separate stress conditions, such as drought or the result of the impairment of the photorespiratory cycle. Detailed transcriptomic analysis showed that the biosynthesis of phenolic compounds was affected in the mutant plants in these two different types of stress situations. For this reason, the genes and metabolites related to this metabolic route were further investigated using a combined approach of gene expression analysis and metabolite profiling. A high induction of the expression of several genes for the biosynthesis of different branches of the phenolic biosynthetic pathway was detected by qRT-PCR. The extent of induction was always higher in *Ljgln2-2*, probably reflecting the higher stress levels present in this genotype. This was paralleled by accumulation of several kaempferol and quercetine glycosides, some of them described for the first time in *L. japonicus*, and of high levels of the isoflavonoid vestitol. The results obtained indicate that the absence of GS_2_ affects different aspects of phenolic metabolism in *L. japonicus* plants in response to stress.

## Introduction

Glutamine synthetase (GS, EC 6.3.1.2) is the key enzyme in charge of glutamine biosynthesis in nature. This enzyme catalyzes the incorporation into one molecule of glutamate of the ammonium derived either from primary nitrogen assimilation (nitrate reduction, N_2_ fixation) or nitrogen reassimilation in the plants. Two types of GS isoenzymes exist in plants: cytosolic (called GS_1_) or plastidic (called GS_2_). GS_1_ is localized in the vascular tissue and plays an important role in the assimilation of external ammonium, the ammonia derived from N_2_ fixation and other sources of nitrogen, as well as in the remobilization of nitrogen during senescence. On the other hand, GS_2_ is predominantly expressed in green tissues, and it has been demonstrated that this particular isoform has an essential role in the reassimilation of the ammonium released by photorespiration and it has also implications in the general metabolism of the plant, and also in nodulation (Betti et al., 2014). The two major isoforms of GS seem to play important roles during stress. GS_1_ has been implicated in the production of proline in the phloem (Brugière et al., 1999) and/or in the remobilization of nitrogen during chronic water stress (Bauer et al., 1997) whereas GS_2_ was also associated with stress tolerance (Kozaki and Takeba, 1996; Hoshida et al., 2000). More recent work of our group has also demonstrated an involvement of GS_2_ in proline production of *L. japonicus* plants in the response to drought stress (Díaz et al., 2010). However, little is known yet on the implications of the different isoforms of GS in relation with other aspects of the stress responses in plants, particularly phenolic metabolism.

Plant phenolics are the most widely distributed secondary metabolites that are involved in the response to stress (Cheynier et al., 2013). This family comprehends a huge array of compounds that arise from phenylalanine with either monomeric or polymeric combinations of the original phenolic ring (for comprehensive reviews of the genetic and biochemistry of plant phenolics metabolism see Davies and Schwinn, 2006 and Saito et al., 2013). Flavonoids are one of the most important classes of plant phenolics in the response to stress, since they respond to almost all kinds of adverse environmental conditions and play several protective roles. Flavonoids can provide protections against UV light and pathogens (Winkel-Shirley, 2002) and exhibit high antioxidative activity *in vitro*, reason why they are also assumed to function as antioxidants *in vivo* (Nakabayashi and Saito, 2015). In agreement with this hypothesis, recent reports showed an increased tolerance to oxidative stress and drought stress in transgenic plants that overaccumulated flavonoids (Nakabayashi et al., 2014). Moreover, isoflavonoids, a distinct class of flavonoids, are also produced by legume plants. These compounds are the typical legume phytoalexins that defend the plant against pathogens, but also participate in the establishment of symbiosis with nitrogen fixing bacteria (Shelton et al., 2012). Therefore, phenolic metabolism is an attractive topic of study in the response to stress, particularly in legume plants.

Our research group has been working for more than 20 years with *Lotus japonicus*, a plant that has been used as a model for the study of several other legumes (Handberg and Stougaard, 1992), and, particularly, of other cultivated Lotus species that are widely used as pastures, and whose productivity is seriously hampered by abiotic stress conditions like drought (Pal'ove-Balang et al., 2014). Recent works have been carried out in our laboratory using functional genomic approaches in order to study the response to different kinds of stress in *L. japonicus*. This has been carried out using both WT plants and a mutant called *Ljgln2-2* that lacks of the plastidic isoform of glutamine synthetase (GS_2_), the enzyme responsible of the reassimilation of the ammonium produced by the photorespiratory cycle. This mutant has been particularly useful in the identification of several stress-responsive genes in *L. japonicus* (Betti et al., 2012). Two different stress situations have been previously analyzed in these mutant plants. On the one hand, the response to drought stress under CO_2_-enriched atmosphere (0.7% CO_2_, where photorespiration is suppressed; Díaz et al., 2010). On the other hand, the response of the mutant plants under non-drought conditions when transferred from a CO_2_-enriched atmosphere to normal CO_2_ conditions (about 0.04% CO_2_) where the photorespiratory cycle is active (Pérez-Delgado et al., 2013). In the case of drought, *Ljgln2-2* accumulated lower levels of proline compared to the WT, a molecule that plays several protective roles under drought and osmotic stress. On the other hand, active photorespiration (PR) produced in *Ljgln2-2* a completely different stress situation due to the impairment of the photorespiratory cycle and the corresponding accumulation of photorespiratory ammonium (Pérez-Delgado et al., 2013). Transcriptomic profiling indicated that these two different stress conditions caused massive transcriptomic changes in the mutant genotype and, to a lower extent, in the WT (Díaz et al., 2010; Pérez-Delgado et al., 2013).

The present work was aimed to investigate if plastidic glutamine synthetase, a key enzyme for nitrogen and photorespiratory metabolism, may also play a role beyond primary metabolism; more specifically, if the lack of this enzyme may affect the response of phenolic metabolism under stress. For this purpose, a comparative transcriptomic analysis of the response of *L. japonicus* WT and *Ljgln2-2* mutant plants to either drought or active PR was carried out. An ample transcriptional modulation of several genes related to phenolic metabolism was observed in these two different types of stress situations. The different genes and metabolites related with the biosynthesis of phenolic compounds, particularly flavonoid and isoflavonoids, have been further analyzed by measurements of gene expression and metabolite profiling.

## Materials and methods

### Plant growth conditions and harvesting of plant material

The model legume *L. japonicus* (Regel) K. Larsen ecotype “Gifu” was used in all the experiments carried out. Seeds were initially obtained from Jens Stougaard (Aarhus University) and self-propagated at the University of Seville. The *Ljgln2-2* mutant that lacks of plastidic GS_2_ protein and activity, was isolated from photorespiratory mutants screening carried out using ethyl methanesulfonate as previously described (Orea et al., 2002). The mutant offspring of two consecutive backcrosses into the WT background were employed. WT and mutant seeds were scarified and surface sterilized and germinated in 1% (w/v) agar in Petri dishes. Later on they were transferred to pots using vermiculite as solid support for the active PR experiment and a mixture of sand:vermiculite (1:1, v/v) for the drought stress experiment. Five seedlings were planted in each pot and grown during 35 days in a growth chamber under 16-h-day (20°C) and 8-h-night (18°C) conditions with a photosynthetic photon flux density of 250 μmol m^−2^ s^−1^ and a constant humidity of 70%. CO_2_ was automatically injected to a final concentration of 0.7% (v/v) to allow normal growth of the *Ljgln2-2* mutant in an atmosphere where PR was suppressed. Plants were irrigated with “Hornum” nutrient solution containing 5 mM NH_4_NO_3_ and 3 mM KNO_3_ (Handberg and Stougaard, 1992). After 35 days of growth under high CO_2_ atmosphere the plants had an average number of 7 trefoils. At this time, total leaf tissue was harvested for each plant genotype, constituting the control condition (zero time) for both the active PR experiments and for the drought stress experiments (see Supplemental Figure S1). For the active PR treatment, plants were then transferred for 2 days from high CO_2_ (0.7% CO_2_ v/v) to normal CO_2_ conditions (0.04% CO_2_ v/v) and harvested, as also described by Pérez-Delgado et al. (2013). For the drought treatment plants were grown all the time under high CO_2_ (0.7% CO_2_ v/v) conditions and drought was imposed by withholding watering for 4 days, after which both WT and *Ljgln2-2* mutant genotypes showed a relative water content of about 55% (see Díaz et al., 2010 for more details), and the plants were subsequently harvested. All the leaf samples used in this work were harvested 4 h after the beginning of the light period. For the active PR treatment, no visible symptoms of the air sensitivity phenotype of the *Ljgln2-2* mutant plants were observed after 2 days under active photorespiratory conditions.

The leaf tissue for RNA extraction was pooled and flash-frozen in liquid N_2_, grinded with a pestle in a mortar that was pre-cooled with liquid N_2_ and the powder was stored at −80°C until use. Leaf tissue for metabolite analysis was dried for 2 days at 100°C using plastic weighing bottles and stored in the dark at room temperature until use. Previous studies showed that incubation at temperatures between 80 and 100°C for long period of time did not change significantly the main flavonoid profiles (Pal'ove-Balang et al., unpublished results), in agreement with previous reports from other groups (Heigl and Franz, 2003). Three independent biological replicates were harvested for each genotype and condition. A biological replicate consisted of tissue pooled from the five plants grown in the same pot.

### RNA extraction and quantitative real-time RT-PCR

Total RNA was isolated from leaf tissues stored at −80°C using the hot borate method (Sánchez et al., 2008). The integrity and concentration of the RNA preparations were checked using an Experion bioanalyzer (Bio-Rad) with RNA StdSens chips and a Nano-Drop 2000 (Nano-Drop Technologies), respectively. For real-time qRT-PCR analysis, total RNA was treated with the TURBO DNA-free DNase (Ambion). Reverse transcription was carried out using the SuperScript III reverse transcriptase (Invitrogen), oligo(dT), and RNAsin RNase inhibitor (Ambion). DNA contamination was checked by carrying out quantitative real-time RT-PCR reactions with oligonucleotides that amplified an intron in the *L. japonicus* Hypernodulation Aberrant Root (*LjHAR1*; chr3.CM0091.1690.r2.m) gene. The efficiency of cDNA synthesis was checked by amplifying the 3′ and 5′ ends of the gene encoding for *L. japonicus* glyceraldehyde-3-P dehydrogenase (*LjGAPDH*; chr4.CM1854.510.r2.a). qRT-PCR reactions were carried out in 10 μL final volume with a 384-well PCR plate in a LightCycler 480 thermal cycler (Roche) using a SensiFAST SYBR No-ROX Kit (Bioline). Expression data were normalized using the geometric mean of three housekeeping genes: *L. japonicus* protein phosphatase 2A (*LjPp2A*; chr2.CM0310.22), *L. japonicus* ubiquitin carrier protein 10 (*LjUbc10*; chr1.TM0487.4), and *L. japonicus* polyubiquitin 4 (*LjUbq4*; chr5.CM0956.27), that were selected among the most stably expressed genes in plants (Czechowski et al., 2004). A list of all the oligonucleotides used is provided in Supplemental Table S1.

### Transcriptomic data analysis

The datasets generated by Díaz et al. (2010) for drought stress and Pérez-Delgado et al. (2013) for active PR were further analyzed in this work. Both transcriptomic experiments were carried out using the Affymetrix GeneChip Lotus1a520343 and the corresponding “Minimum Information about a Microarray Experiment” (MIAME)-compliant data are deposited at Array Express with the accession codes E-MEXP-2690 and E-MEXP-3603 for the drought and active PR experiments respectively.

The differentially expressed genes between control conditions and stress treatments were identified by the analysis of the different Affychip gene probesets using a significance-based comparison at a false discovery rate (FDR) of < 0.05. In the *L. japonicus* Affymetrix GeneChip Lotus1a520343 several gene probesets may recognize the same gene. For this reason the number of gene probesets that changes under each comparison may be higher than the real number of genes that are actually modulated in the plant. The different gene probesets that changed in the different comparisons were visualized using the MapMan program (Usadel et al., 2005) and analyzed according to the corresponding metabolic pathways. Since different probesets from the gene chip may recognize the same transcript, homology search was carried out using the Blast program at the Kazusa database (http://www.kazusa.or.jp/lotus/) in order to assign with more accuracy each probeset to the corresponding gene. For the determination of the significantly overrepresented pathways within a group of gene probesets the Pathexpress software was used (Goffard and Weiller, 2007) with a statistical cut-off of *p* < 0.05.

### Metabolites profiling analysis

For analytical HPLC analysis, phenolic compounds were extracted from 50 mg of dry leaves with 1 ml of 50% (v/v) methanol or 1 ml of 100% (v/v) methanol (for determination of isoflavonoids and tannins), spinned shortly and filtered using a 0.2 μm membrane. Acid hydrolysis was performed for 15 min at 95°C in a water bath after addition of HCl 2.9 N (volume ratio of the methanol extract to HCl 1:1). Both the hydrolyzed and non-hydrolyzed samples were analyzed by gradient reversed phase high-performance liquid chromatography (HPLC) on Agilent 1260 Infinity Quaternary LC System with 1260 Infinity DAD detector and Kromasil C_18_ 250 × 5 μm I. D. reversed phase column. The mobile phases were 5% acetonitrile (A) and 90% acetonitrile (B). The solvent was delivered to the column at a flow rate of 0.7 ml/min as follows: 0 min, A/B (75:25); 0–25 min, linear gradient to A/B (50:50); 25–30 min, linear gradient to A/B (0:100); 30–40 min A/B (0:100); 40–50 min, linear gradient to A/B (75:25); detection at 280 and 370 nm. For the analysis of vestitol the mobile phases were 60% acetonitrile (A) and 90% acetonitrile (B) and the gradient protocol was 0–5 min, A/B (100:0); 5–15 min, linear gradient to A/B (50:50); 15–20 linear gradient to A/B (0:100); 20–25 min A/B (0:100); 25–30 linear gradient to A/B (100:0).

The peaks were identified based on their retention times and UV-VIS spectra measurements carried out during the analysis in comparison to commercially available standards of quercetine, kaempferol, p-coumaric acid, p-ferulic acid (Sigma-Aldrich) and upon the data of Suzuki et al. (2008) and Lanot and Morris (2005). LC/ESI-MS analyses of flavonoid glycosides were carried out on Dionex UltiMate 3000 Quarternary Analytical LC System with diode array detector (Germering, Germany) interfaced to a Varian 310 MS, Triple Quadrupole mass spectrometer with electrospray ionization (ESI) source (Walnut Creek, CA, USA). The source parameters were as follows: positive-ion mode, capillary voltage of 4 kV, nebulization with nitrogen at 50.0 psi, drying with nitrogen at 30.0 psi and 300°C. Mass spectra were recorded between *m/z* 200 and 1000.

For total tannin measurements, methanol extracts were centrifuged for 5 min at 3000 × g and then 1 ml aliquots of supernatant were assayed with 5 ml of 1% vanillin containing 1.2 N HCl in absolute methanol. Each sample was left in a water bath at 30°C for exactly 20 min together with the corresponding blank containing 1 ml of supernatant and 5 ml 1.2 N HCl (without vanillin). Absorbance was registered at 500 nm and calculated to catechin equivalents.

In order to collect enough material to unambiguously identify compounds by NMR, kaempferol-glycosides were isolated by combining glass column chromatography and preparative HPLC. Dry tissue from plants leaves (60 g approximately) grown under control conditions was subsequently extracted with chloroform, methanol, 50% methanol, and finally with acetic acid. The methanol extracts were shaken 3 times with chloroform to remove the rest of chlorophylls, the methanol and chloroform layer was separated each time by addition of some drops of distilled water. Glass column (90 cm long, 5.5 cm in diameter) chromatography on silica gel was used for both methanol and 50% methanol fractions with chloroform–methanol (20:1–5:2) solution as a mobile phase. About 400 fractions were collected by automatic collector, 15 ml of each. The compounds in fractions were followed during the glass-column separation by analytical HPLC (in the same analytical conditions as described above for crude extract analyses). The step-by step changes of chloroform–methanol ratio in mobile phase in the glass column was decided according to the flavonoid content of the collected fractions. Fractions were joined according to their content, evaporated in Rotation vacuum evaporator and re-dissolved in methanol. The abundant peaks were then further purified using preparative HPLC system consisting of a Tessek SGX C18 column (7 μm 8 × 250 mm), a ECOM LCP 4100 pump and a UV-VIS detector LCD 2040 and a flow rate of 4 ml/min, to be suitable for NMR analysis. The compounds identities were verified by NMR spectra at room temperature on NMR spectrometer Varian VNMRS 600 (Palo Alto, CA, USA) operating at 599.868 MHz for ^1^H and 150.836 MHz for ^13^C. Spectra were recorded in CD_3_OD-d_4_. The 2D gCOSY, 290 TOCSY, NOESY, gHSQC, gHMBC (optimized for a long-range coupling of 8 Hz), 1D291 sel TOCSY, gH2BC, and gTOXY-HSQC methods were employed.

### Statistical data analysis

Significant changes in gene expression levels in the microarray experiments were analyzed using a significance-based comparison applying a false discovery rate (FRD) < 0.05. Other details as described by Pérez-Delgado et al. (2013). Significant differences in gene expression levels (qRT-PCR) between control and stress conditions were determined for each genotype according to Student's *t*-test (*p* < 0.05). Data from the metabolite measurements were analyzed according to the Student's *t*-test with *p* < 0.05 or *p* < 0.01.

## Results

### Comparative transcriptomic analysis of the response of *L. japonicus* to drought and active photorespiration

In order to investigate the role of plastidic GS_2_ in the response to stress in *L. japonicus*, a mutant called *Ljgln2-2* that lacks of this enzyme was submitted to two different stress treatments: drought or active PR. The gene probesets significantly modulated by each stress treatment in leaves of the mutant plants were identified by comparing the transcriptome obtained under stress conditions with the transcriptome of the plants in control conditions (normal watering and suppressed PR). For comparative purposes, WT plants were also submitted to the same treatments. In total, 2608 and 1480 gene probesets were modulated by drought or active PR respectively in the WT compared to the control conditions, while 7915 and 6610 gene probesets changed in the mutant plants under the same conditions (Table 1). Interestingly, 2173 gene probesets were commonly affected by both types of stress situations in *Ljgln2-2*, which corresponded to 27.5 and 41.0% of the total number of gene probesets modulated by drought or active PR respectively (Table 1). This suggested the existence of a common response to different stress conditions in the *Ljgln2-2* mutant. In contrast, in the case of the WT, only 187 gene probesets were found to be commonly modulated in drought or active PR (Table 1). The genes that were commonly modulated in the response to drought or active PR are further analyzed below in this paper separately for WT and the *Ljgln2-2* mutant plants.

**Table 1:** **Total number of gene probesets modulated by drought stress or active PR in WT and ***Ljgln2-2*** mutant plants**.

	**Experiment 1 Drought**	**Experiment 2 Active PR**	**Commonly affected**
WT	2608	1480	187
*Ljgln2-2*	7915	6610	2173

*The total number of gene probesets that changed significantly (p < 0.05) by drought was calculated by comparing the gene expression levels under drought conditions with the expression levels under control conditions (high CO_2_ atmosphere, normal watering) as described by Díaz et al. (2010). The gene probesets that changed significantly (p < 0.05) by active PR conditions were identified by comparing the gene expression levels with a control under photorespiratory suppressed conditions (high CO_2_ atmosphere, normal watering) as described by Pérez-Delgado et al. (2013). The column on the right shows the total number of gene probesets that were commonly elicited in the drought stress experiment and in the active PR experiment*.

### Analysis of the gene probesets modulated by drought or active PR

The 187 gene probesets that changed under the two types of stress situation in WT plants were represented in the context of general metabolism using the MapMan software (Supplemental Figure S2, see Supplemental Table S2 for a list of these gene probesets). The MapMan program allows the visualization of the changes observed in transcriptomic data by providing an overview of metabolic pathway responses. Each significantly modulated gene is indicated with a square within a box that represents the corresponding metabolic pathway. MapMan visualization indicated that the genes commonly affected by drought or active PR in the WT were quite dispersed through the metabolic map and no particular pathway seemed to be highly regulated. In contrast, some interesting correlations were observed in the analysis the group of 2173 gene probesets that were commonly modulated by drought or active PR in *Ljgln2-2* mutant plants. First of all, it was shown that the great majority of these gene probesets (2030) changed in the same direction (i.e., were commonly induced or repressed) in response to either drought or active PR (Supplemental Figure S3). Of these, 1073 gene probesets were commonly induced while 957 were commonly repressed. Linear regression analysis indicated that the gene probesets that changed in the same direction under both types of stress conditions were slightly more modulated by drought compared to active PR (Supplemental Figure S3).

**Figure 1:**
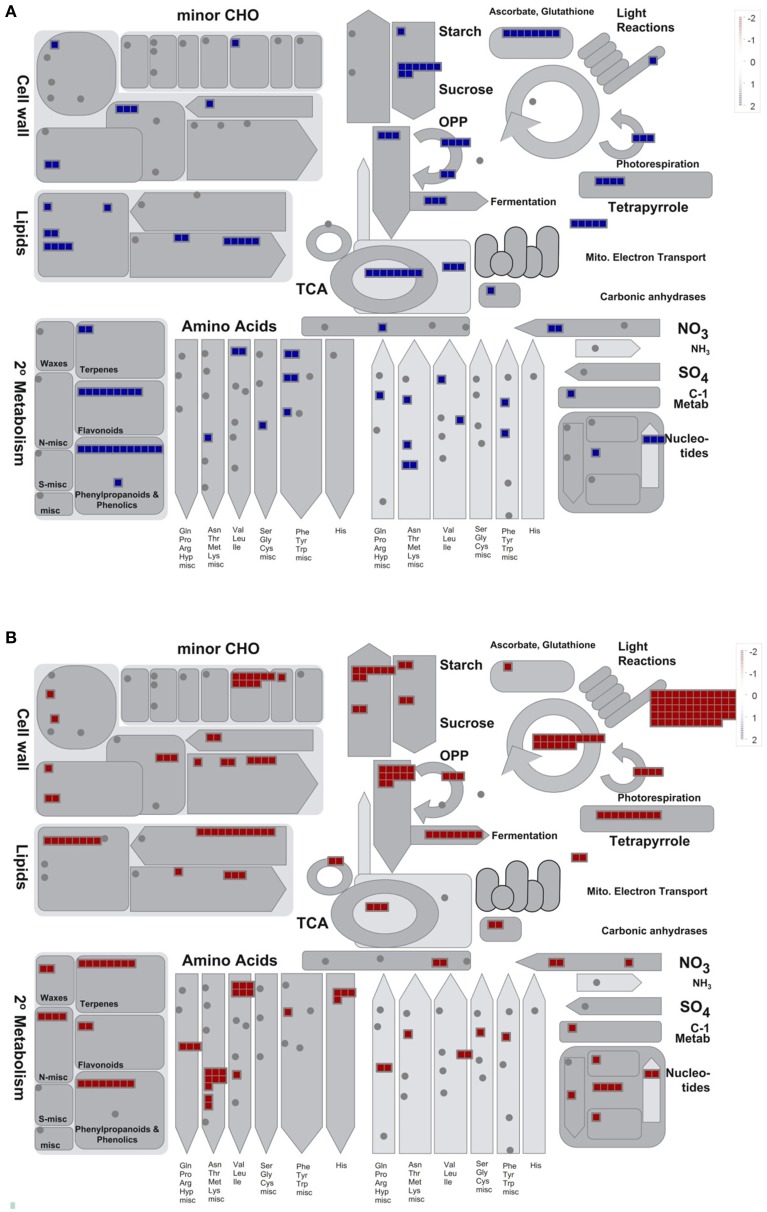
**MapMan overview of general metabolism for the gene probesets that were commonly induced or repressed in ***Ljgln2-2*** mutant plants either in drought or in active PR stress conditions. (A)** Shows the 1073 gene probesets that were commonly induced by the two types of stress situations and **(B)** the 957 gene probesets that were commonly repressed analyzed in the context of general metabolism using the MapMan software. Each square represent a gene probeset that changed significantly (*p* < 0.05) under both types of stress situation. The gene probesets are within a figure that represents the correspondent metabolic pathway, in the case of cyclic pathways like the TCA cycle (the circle in the center of the figure) and the Calvin cycle (the circle on the upper-right side of the figure) the pathway is represented with a circle. The arrow-shaped pathways (for example for the amino acids, the different arrows at the bottom of the figure) represent routes that supply or deplete of intermediates another cycle. For example in the case of amino acid metabolism the arrows that point up to the TCA cycle represent amino acid degradation (that replenish TCA of intermediates) while the ones that point down are the routes for their biosynthesis, that deplete TCA of intermediates. More details can be found in Usadel et al. (2005). An arbitrary fold-change value of 2 and −2 was assigned to the gene probesets that were induced and repressed respectively since their extent of induction or repression was different under either drought or active PR. Although these gene probesets changed in the same direction under the two types of stress situations analyzed, the extent of their changes was different under each treatment; for this reason it was not possible to report in the same graph the level of fold-change under either drought or active PR. Not all the gene probesets considered in the text (the 1073 induced and the 957 repressed ones) were shown in this figure since several categories of gene products like transporters, transcription factors, protein kinases etc… are not represented in the MapMan overview of general metabolism that is visualized in here.

In order to get further insight into the convergent response of *Ljgln2-2* to drought and active PR, the 1073 gene probesets that were commonly induced by drought or active PR treatments were visualized in the context of general metabolism using the MapMan software (Figure 1A). The 957 gene probesets that were repressed by both types of stress are represented in Figure 1B. Since the extent of change for these commonly induced and repressed gene probesets was different under either drought or active PR, a fold-change value of 2 and -2 was arbitrarily assigned for the MapMan representation of Figure 1. Several genes related to ascorbate and glutathione metabolism, sucrose degradation and phenylpropanoid metabolism were highly induced by both stress treatments (Figure 1A). On the other hand, the analysis of the genes commonly repressed by these two types of stress treatments showed a general repression of several pathways related to photosynthesis like the biosynthesis of the structural components of the photosystems, the Calvin cycle, photorespiration and genes related to carbohydrate degradation (Figure 1B). To determine which metabolic pathways were significantly over-represented among these groups of genes, the common gene probesets induced or repressed under these two types of stress treatments were further analyzed using Pathexpress (Goffard and Weiller, 2007). Pathexpress is a web-based tool that allows the identification of the most relevant sub-networks (metabolic pathways, sub-pathways, and enzyme neighborhood) associated with a set number of genes. An over-represented pathway in a query list of gene probesets is identified by comparing the number of enzymes reactions of this route represented within the query list compared to the total number of enzyme reactions present in the pathway. The significance of this comparison is then tested using a hypergeometric distribution test as developed in the BlastSets system (Goffard and Weiller, 2007 and references therein). The analysis carried out confirmed that the most significantly repressed pathways were mainly related to photosynthesis and carbon metabolism. Among the most repressed routes were glycolysis, starch and sucrose metabolism, carbon fixation (essentially the Calvin cycle) as well as the biosynthesis of chlorophyll and photosynthetic pigments (Table 2). On the other hand, the most induced pathway by both stress treatments in the *Ljgln2-2* mutant was stilbene/lignin/coumarin biosynthesis, which comprises several branches of the complex route for the biosynthesis of phenolic compounds (Table 2). Flavonoid biosynthesis, another branch of plant phenolics metabolism, was also found among the most over-represented routes, as well as phenylalanine metabolism, where genes encoding for the enzymes for the first steps of the biosynthesis of phenolic compounds like phenylalanine ammonia lyase (PAL) and cinnamate 4-hydroxylase (C4H) were induced. In addition, it was also noted that one particular gene for phenolic metabolism, corresponding to isoflavone 2′-hydroxylase, was among the top 20 commonly induced genes under drought stress conditions in the *Ljgln2-2* mutants, and was the most induced genes by active PR in this genotype (Supplemental Table S3).

**Table 2:** **Pathexpress analysis of over-represented pathways among the gene probesets that were commonly induced or repressed by drought or active PR treatments in the ***Ljgln2-2*** mutant**.

**Pathway**	**Nb. of enzymes in the pathway**	**Nb. of enzymes in the dataset**	***P*-value**
**INDUCED**			
Stilbene, coumarine and lignin biosynthesis	10	7	0.0001
Lysine degradation	12	7	0.0005
Aminosugars metabolism	12	6	0.0039
Flavonoid biosynthesis	15	6	0.0144
Ascorbate and aldarate metabolism	8	4	0.0194
Butanoate metabolism	21	7	0.0243
Methane metabolism	9	4	0.0311
Glycerolipid metabolism	18	6	0.0368
Phenylalanine metabolism	14	5	0.0421
Ether lipid metabolism	10	4	0.0460
Lipopolysaccharide biosynthesis	10	4	0.0460
Sphingolipid metabolism	10	4	0.0460
**REPRESSED**			
Glycolysis/Gluconeogenesis	27	13	0.0005
Histidine metabolism	16	9	0.0010
Fructose and mannose metabolism	23	11	0.0015
Starch and sucrose metabolism	31	13	0.0025
Carbon fixation	22	10	0.0040
Tetrachloroethene degradation	3	3	0.0071
Novobiocin biosynthesis	3	3	0.0071
Ascorbate and aldarate metabolism	8	5	0.0085
Lysine degradation	12	6	0.0155
Metabolism of xenobiotics by cytochrome P450	4	3	0.0242
Porphyrin and chlorophyll metabolism	20	8	0.0242
Fatty acid biosynthesis	7	4	0.0288
Pentose and glucoronate interconversions	11	5	0.0426

*The significantly over-represented pathways (P < 0.05, no correction) were identified using the Pathexpress software among the groups of gene probesets that were commonly induced or repressed by drought or active PR. The total number of enzymes that compose a pathway (according to the KEGG database) and the number of enzymes of this pathway present in the set of gene probesets analyzed are reported, as well as the significance of the comparison between these two numbers*.

Previous works have analyzed the consequences of the lack of GS_2_ in primary metabolism (Pérez-Delgado et al., 2013). Interestingly, the results obtained now in the present paper indicated that the biosynthesis of phenolic compounds was also greatly modulated in response to stress as a result of the absence of plastidic GS_2_ in the *Ljgln2-2* mutant. The following sections of this paper analyze further in detail by qRT-PCR and metabolic profiling the different changes in phenolic metabolism associated with the lack of GS_2_ in response to drought or active PR stress conditions.

### Modulation of the expression of gene probesets for the biosynthesis of phenolic compounds

A real-time qRT-PCR analysis was carried out to further investigate and validate the changes in the expression of genes for the biosynthesis of phenolic compounds that were found in the transcriptomics studies. Given the great complexity of the biosynthetic route of plant phenolics a focus was made on the key genes encoding for enzymes that catalyze the first common steps of the route, and also several ones for the central flavonoid biosynthetic pathway, for isoflavonoid biosynthesis as well as for the branch that lead to anthocyanins and protoanthocyanidins. Several of the enzyme activities of this pathway are encoded by large gene families like in the case of phenylalanine-ammonia lyase, encoded by 10 genes, and chalcone synthase encoded by 12 (Supplemental Table S4). Moreover, some of these gene families were organized in tandem repeats on the same chromosome like in the case of chalcones isomerase (Shimada et al., 2003), dihydroflavonol reductase (Shimada et al., 2005), and several genes for isoflavonoids biosynthesis (Shimada et al., 2007 and this work, Supplemental Table S4). Given the high number of genes that compose this route and the aforementioned gene redundancy, a set of specific oligonucleotides were utilized that amplified only specific copies of the redundant gene probesets (Supplemental Table S1). In addition, when multiple gene copies were present, only the transcripts that changed under stress according to the transcriptomic analysis were considered (see Supplemental Table S1).

**Figure 2:**
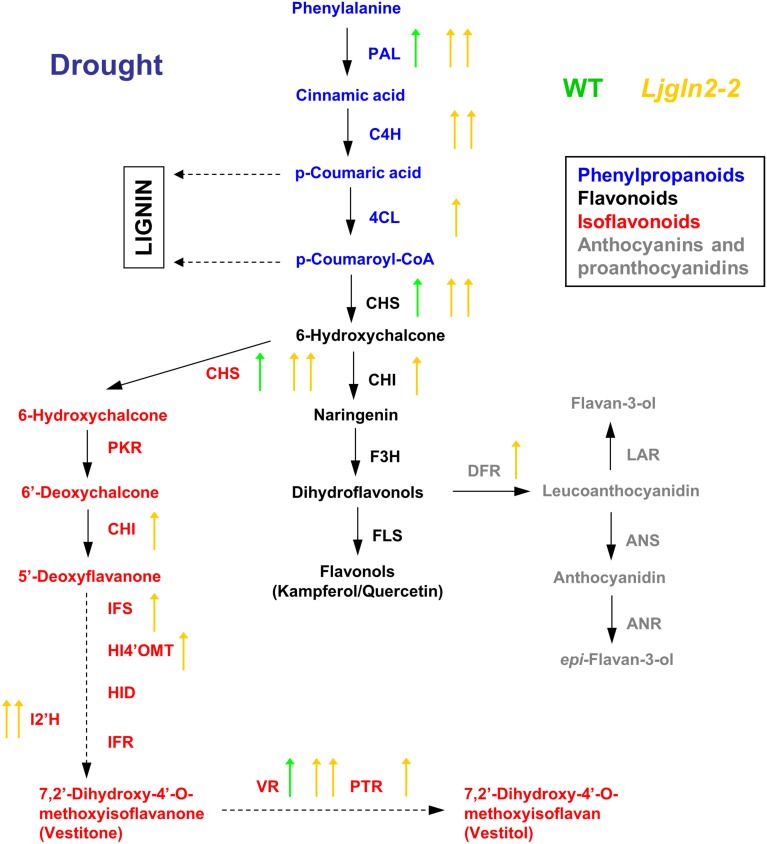
**Graphical summary of the changes in the expression of genes for the biosynthesis of phenolic compounds under drought conditions**. qRT-PCR data are from Table 3. Arrows highlight significant changes in gene expression according to the analysis of Table 1; green and yellow represent WT and *Ljgln2-2* plants respectively. Arrows pointing up mean induction and arrows pointing down mean repression; one arrow means moderate (<6 times, up or down) modulation while two arrows mean high (>6 times, up or down) modulation. A color code has been used to emphasize different branches of the pathway: blue for the common initial steps for phenylpropanoid biosynthesis, black for some enzymes of the central flavonoid biosynthetic pathway, gray for some enzymes for anthocyanins and protoanthocyanidins and red for some enzymes for isoflavonoid biosynthesis. For space reasons several intermediates in the biosynthesis of isoflavonoids have been omitted. The genes measured encode for enzymes that belongs to: (1) the common “entry” to the pathway, that comprehends the genes encoding for PAL, C4H and 4CL; (2) the central flavonoid biosynthetic pathways including the two key enzymes for the formation of the cyclic flavonoid scaffold: CHS, CHI, F3H, and FLS for the branch that leads to flavonols; (3) the first committed reactions for the biosynthesis of anthocyanins: DFR and ANS and for the biosynthesis of protoanthocyanidins (also known as condensed tannins): LAR and ANR; 4) the branch for the biosynthesis of isoflavonoids, that shares the enzyme activities CHS and CHI with the common flavonoid pathway, but also needs the activities of PKR, IFS, HI4'OMT, HID and I2'H and the “late” activities of this branch that lead to the formation of the phytoalexins vestitone: IFR and of the isoflavan vestitol: VR and PTR. For a detailed description of these steps see Davies and Schwinn (2006) and Shelton et al. (2012).

**Figure 3:**
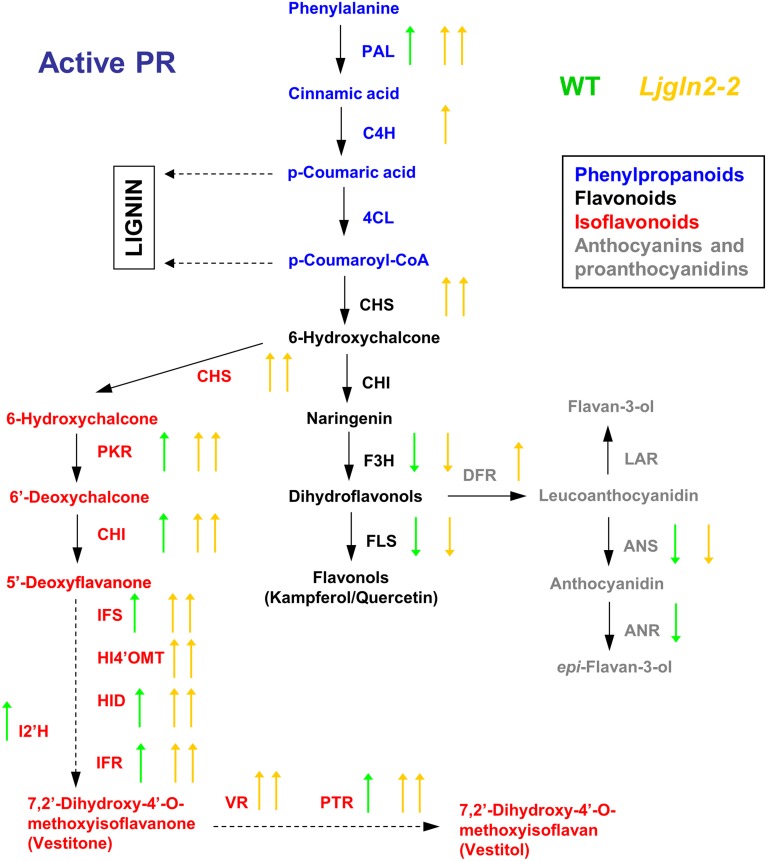
**Graphical summary of the changes in the expression of genes for the biosynthesis of phenolic compounds under active photorespiratory conditions**. Other details as in the legend of Figure 2.

A global overview of the results obtained from qRT-PCR measurements of the different genes analyzed are shown separately for the WT and Ljgln2-2 mutant plants that were submitted to either drought stress (Figure 2) or active PR (Figure 3). The data obtained confirmed that different branches of phenolic metabolism were highly modulated either in the drought or active PR treatments, especially in the mutant genotype. Table 3 provides additional quantitative data obtained for the most representative genes of the phenolic biosynthetic pathway (see the legend of this Table for the abbreviation of the enzymes of the route). Supplemental Figure S4 shows that the fold-change in expression levels obtained for the selected genes analyzed by qRT-PCR was in good agreement with the microarray data previously analyzed. Further analysis of qRT-PCR data indicate that the common enzymes of the phenolic biosynthetic pathway (PAL, C4H, and 4CL) were highly induced in drought or active PR treatments particularly in the mutant plants. Moreover, PAL, the entry-point enzyme of the route was also induced in the WT genotype by both types of stress conditions. Since PAL plays a key role in mediating carbon flux into the phenolic pathway (Zhang and Liu, 2015), these data suggested increased biosynthesis of phenolic compounds in both WT and mutant genotypes. CHS and CHI, the enzymes that create the common scaffold of flavonoids and isoflavonoids, were also highly induced by both treatments especially in the mutant. Interestingly, F3H and FLS, which produce the precursor for flavonol biosynthesis, were repressed by active PR in both genotypes. Regarding the key genes for anthocyanins and protoanthocyanidins biosynthesis, DFR (that catalyzes the first step of these two branches of the route) was induced exclusively in the mutant by both treatments, while ANS and LAR (that produce respectively anthocyanidins and flavan-3-ols, both precursors of condensed tannins) were repressed by active PR in both genotypes. Finally, several genes for isoflavonoids biosynthesis were induced under the two types of stress conditions that were examined. This induction was dramatic in the case of active PR, where all the genes for isoflavonoid biosynthesis were induced at least 9 times in the mutants and several of them were also induced, although to a lesser extent, in the WT. On the other hand, different genes for isoflavonoid biosynthesis were also induced by drought stress but exclusively in the mutant genotype.

**Table 3:** **Changes in the relative expression levels of selected genes for the biosynthesis of phenolic compounds measured by qRT-PCR**.

	**Fold change in gene expression**			
	**Drought**		**Active photorespiration**	
	**WT**	***Ljgln2-2***	**WT**	***Ljgln2-2***
**GENE PRODUCT**				
PAL	3.06 ± 1.15	7.86 ± 2.43	1.90 ± 0.59	10.78 ± 2.50
C4H	2.39±0.78	10.7 ± 4.72	1.21±1.16	16.12 ± 3.22
4CL	1.09±0.62	5.14 ± 1.67	1.29±0.41	1.93 ± 0.79
CHS	3.76 ± 0.45	9.59 ± 2.04	2.54±1.70	13.85 ± 5.12
CHI	1.52±0.51	2.66 ± 0.65	2.02 ± 0.40	9.65 ± 2.53
F3H	0.86±0.36	0.68±0.33	0.58 ± 0.08	0.38 ± 0.09
FLS	0.74±0.40	0.78±0.40	0.63 ± 0.02	0.55 ± 0.01
DFR	1.70±1.12	3.21 ± 1.63	0.83±0.20	8.16 ± 1.34
ANS	1.16±0.39	1.28±0.58	0.27 ± 0.15	0.41 ± 0.18
LAR	1.06±0.21	0.87±031	0.40 ± 0.22	0.36 ± 0.24
ANR	1.21±1.03	1.34±0.96	0.26 ± 0.19	0.71 ± 0.33
PKR	1.16±0.77	1.75±0.44	2.68±1.58	13.04 ± 3.30
IFS	1.59±0.64	5.06 ± 2.13	2.11 ± 0.49	8.85 ± 2.45
HI4′OMT	1.17±0.72	2.08 ± 0.42	1.87±1.22	13.42 ± 6.61
HID	1.38±1.01	1.64±1.10	1.98 ± 0.69	13.67 ± 5.74
I2′H	1.26±0.60	8.16 ± 3.12	3.24 ± 0.79	17.47 ± 7.16
IFR	1.69±0.98	2.50±0.65	1.19±0.38	28.99 ± 17.53
VR	2.95 ± 1.54	18.82 ± 7.49	2.50 ± 0.89	16.38 ± 9.98
PTR	1.85±0.86	2.94 ± 1.01	3.32 ± 1.04	22.25 ± 11.32

*The levels of transcript for each gene under control conditions in each genotype were taken as 1.00. Numbers in red mean significant difference between control and stress conditions for each genotype according to Student's t test (p < 0.05). Transcript levels have been measured for three independent biological replicates. The gene products highlighted in blue belong to the entry of the pathway, black highlights the enzymes of the central flavonoid biosynthetic pathway, gray highlights enzymes for anthocyanins and protoanthocyanidins biosynthesis and in red are highlighted enzymes for isoflavonoid biosynthesis. The abbreviations for the enzyme activities are: PAL, phenylalanine-ammonia lyase; 4CL, 4-Coumarate:CoA ligase; C4H, cinnamate-4-dehydrogenase; CHS, chalcone synthase; CHI, chalcone isomerase; F3H, flavanone 3β-hydroxylase; FLS, flavonol synthase; DFR, dihydroflavonol reductase; ANS, anthocyanin synthase; LAR, leucoanthocyanidin reductase; ANR, anthocyanidin reductase; PKR, polyketide reductase also called chalcone reductase; IFS, isoflavone synthase; HI4'OMT, 2,4,7′-Hydroxyisoflavanone 4′-O-methyltransferase; HID, isoflavanone dehydratase; I2′H, isoflavone 2′-hydroxylase; IFR, isoflavone reductase; VR, vestitone reductase; and PTR, pterocarpan reductase. CHS and CHI are reported as belonging to the flavonoid biosynthetic pathway but are also fundamental for the biosynthesis of isoflavonoids. Expression data were normalized using the geometric mean of three housekeeping genes: L. japonicus protein phosphatase 2A (LjPp2A; chr2.CM0310.22), L. japonicus ubiquitin carrier protein 10 (LjUbc10; chr1.TM0487.4), and L. japonicus polyubiquitin 4 (LjUbq4; chr5.CM0956.27), that were selected among the most stably expressed genes in plants (Czechowski et al., 2004). Data are the mean ± S.D. of three independent biological replicates*.

Taken together, these results strongly suggest that the two types of stress situations analyzed in this paper may stimulate in different ways the production of phenolic compounds, particularly in the Ljgln2-2 mutant genotype. Noteworthy, a much higher induction of specific genes for isoflavonoid biosynthesis was observed in the mutant plants compared to the WT, particularly under photorespiratory active conditions.

### Metabolite profiling of phenolic compounds under control and stress conditions

**Figure 4:**
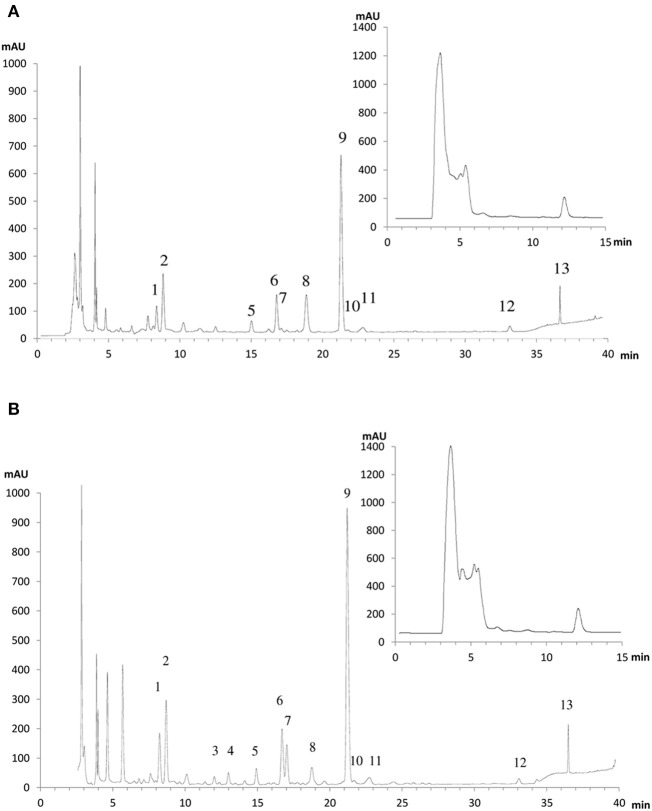
**HPLC elution profiles of flavonoids and isoflavonoidsfrom ***L. japonicus*** leaves of (A) WT plants under normal watering conditions and (B) WT plants under drought stress conditions**. A typical chromatographic profile for 50% methanol leaf extracts from WT plants grown under either control conditions or drought stress conditions is presented. The compounds were detected according to their absorbance at 280 nm. The peak numbers correspond to the metabolites: 1: kaempferol-3-O-glucosyl (1-2)-glucoside-7-O-rhamnoside; 2: kaempferol-3-O-glucosyl (1-2)-galactoside-7-O-rhamnoside; 3: quercetin 6-deoxyhexose-hexose; 4: quercetin 6-deoxyhexose-hexose; 5: kaempferol-3-O-galactosyl-7-O-rhamnoside; 6: kaempferol-3-O-glucosyl-7-O-rhamnoside; 7: quercetin 6-deoxyhexose-6-deoxyhexose; 8: p-coumaric acid; 9: kaempferol-3,7-di-O-rhamnoside; 10: p-ferulic acid; 11: simple phenylpropanoid; 12: kaempferol-6-deoxyhexose; 13: flavonol-6-deoxyhexose. The chromatograms shown here are for only one of the six different biological replicates used in this work. For the relative quantification of metabolite levels presented in **Table 5** the mean of all the six replicates was used. In the inset is the chromatographic profile for the 100% methanol leaf extracts, where the peak corresponding to vestitol can be seen, eluting at min 12.

**Figure 5:**
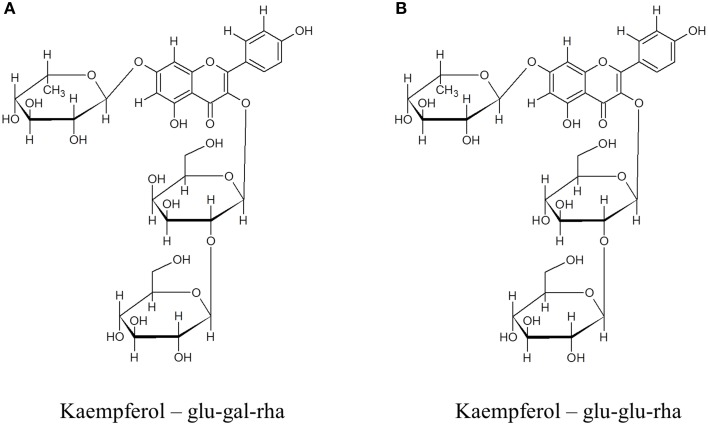
**Chemical structures of (A) kaempferol 3-O-glucosyl (1-2)-galactoside-7-O-rhamnoside and (B) kaempferol 3-O-glucosyl (1-2)-glucoside-7-O-rhamnoside**. The compounds were purified from crude extracts of WT *L. japonicus* leaves from plants grown under control conditions by glass column chromatography followed by preparative HPLC. NMR determination of the chemical structure of the compounds was carried out as described in materials and methods.

To further explore the possible changes in phenolic metabolism in WT and Ljgln2-2 mutants under stress conditions a metabolic profiling of phenolic compounds was carried out in leaves from L. japonicus plants. In order to show the different metabolites identified, we present first, as an example, the analysis carried out by analytical HPLC for WT plants under control conditions and under drought stress conditions (Figure 4). Several peaks corresponding to phenolic metabolites were detected in the chromatograms obtained, which was in good agreement with previous reports from L. japonicus leaves (Suzuki et al., 2008). The analysis carried out allowed detecting all classes of phenolic compounds that may play a role in the response to stress: phenylpropanoids, flavonoids, isoflavonoids, and tannins, while anthocyanins were undetectable. The identity of the compounds was assigned based on their retention times and UV-VIS spectra and confirmed by LC/ESI-MS detection (Supplemental Table S5). Moreover, preparative HPLC was also used in order to purify the most abundant compounds of unknown chemical structure for their identification by NMR. A description of the NMR spectra and of their interpretation can be found online (Supplemental Figure S5). The most abundant flavonoids detected were kaempferol and quercetin, both belonging to the family of flavonols. Total kaempferol and quercetin levels were determined by removing the sugar moieties from the flavonoid scaffold by acid hydrolysis. On the other hand, metabolite profiling without acid hydrolysis permitted the detection of several different kaempferol and quercetin glycosides (Figure 4). Two triple glycosides were found for the first time in L. japonicus leaves: kaempferol 3-O-glucosyl (1-2)-glucoside-7-O-rhamnoside and kaempferol 3-O-glucosyl (1-2)-galactoside-7-O-rhamnoside (Figure 5). Moreover, this is, to our knowledge, the first ever detection of kaempferol 3-O-glucosyl (1-2)-galactoside-7-O-rhamnoside in plants.

Interestingly, some differences in the basal levels of phenolic compounds were detected in control (non-stress) conditions between the WT and the Ljgln2-2 mutant plants (Table 4). The levels of kaempferol glycosides were generally lower in the mutant compared to the WT while quercetin glycosides tended to be higher in the mutant; however these differences were not usually higher than 2-fold (Table 4). These data indicate that the absence of plastidic GS_2_ has some minor effects in phenolic metabolism in L. japonicus plants under non-stress conditions.

**Table 4:** **Relative levels of phenolic compounds in WT and ***Ljgln2-2*** leaves under control conditions (normal watering and suppressed PR)**.

**Compound**	***Ljgln 2-2/WT***
kaempferol-3,7-di-O-rhamnoside^a^^,^ ^b^	0.82±0.28
kaempferol-3-O-glucosyl-7-O-rhamnoside^a^^,^ ^b^	0.65±0.20^*^
kaempferol-3-O-galactosyl-7-O-rhamnoside^b^	0.70±0.23^*^
kaempferol-3-O-glucosyl (1-2)-galactoside-7-O-rhamnoside^a^^,^ ^b^	0.69±0.23^*^
kaempferol-3-O-glucosyl (1-2)-glucoside-7-O-rhamnoside^a^^,^ ^b^	1.10±0.62
*kaempferol-6-deoxyhexose^b^*	0.56±0.33^*^
Total kaempferol (after acid hydrolysis)	0.97±0.34
*Quercetin-6-deoxyhexose, -hexose^b^*	2.46±1.53^*^
*Quercetin-6-deoxyhexose, -hexose^b^*	1.57±1.10
*Quercetin-6-deoxyhexose, 6-deoxyhexose^b^*	1.44±0.98
total quercetine (after acid hydrolysis)	1.87±0.91^*^
*flavonol-6-deoxyhexose^b^*	0.60±0.25^*^
p-coumaric acid	0.64±0.41
p-ferulic acid	0.94±0.51
*Simple phenylpropanoid*	0.76±0.48
vestitol	1.12±0.34
Total tannins	1.05±0.15

The differences in the relative levels of phenolic compounds between WT and Ljgln2-2 mutant plants are reported as the ratio between metabolite levels in Ljgln2-2 and the levels of the same metabolite in WT plants. Total crude extracts from leaves were obtained in 50% methanol and analyzed by HPLC as described in materials and methods, with the exception of vestitol, that due to its low polarity was quantified in leaf extracts obtained in 100% methanol. The different compounds were identified in HPLC chromatograms employing DAD detector. Tannins were determined spectrophotometrically. Identities of compounds were verified by

a NMR or

b *LC/ESI-MS*.

For the compounds in italics it was not possible to determine their accurate chemical structure (e.g., for the two different quercetin-6-deoxyhexose,-hexoses isomers, that showed different retention times). Significant difference between levels in mutant and WT at

* *p < 0.05 (n = 4) according to Student's t-test*.

*Data are the mean ± S.D. of six independent biological replicates*.

Very remarkable changes in the levels of phenolic compounds were observed among WT and Ljgln2-2 mutant plants submitted to either drought or active PR conditions (Table 5). Representative elution profiles for WT under control and drought stress conditions are presented in Figure 4, while the elution profiles for WT under active PR and for the mutant plants under both types of stress situations are available online (Supplemental Figures S6–S8). The total kaempferol content was significantly increased in both genotypes by drought stress but not by active PR. Among the most abundant kaempferol-glycosides there was a significant increase in kaempferol-3,7-di-O-rhamnoside and of the triple glycoside kaempferol-3-O-glucosyl (1-2)-glucoside-7-O-rhamnoside in drought conditions in both genotypes, while no significant changes in total kaempferol or in specific kaempferol-glycosides were observed under active photorespiratory conditions. On the other hand, changes in quercetin levels were also observed under the two different stress conditions examined (Table 5). Total quercetin and different quercetin glycosides were dramatically increased in WT plants under drought stress but not on active PR. In addition, significant, although lower changes in total quercetin and in some quercetin glycosides were observed under both stress conditions in the case of Ljgln2-2. A third, minor flavonol-like 6-deoxyglycoside was also found in leaves, and its levels did not change in any of the conditions considered. p-coumaric acid, the product of the C4H enzyme, was substantially reduced by drought in the WT genotype. Therefore, under the same conditions massive increases in quercetin levels, as well as a smaller increase in kaempferol, were observed for WT plants.

**Table 5:** **Relative changes of phenolic compounds under drought or active photorespiratory conditions**.

**Compound**	**Drought**		**Active photorespiration**	
	**WT**	***Ljgln 2-2***	**WT**	***Ljgln 2-2***
kaempferol-3,7-di-O-rhamnoside^a^^,^ ^b^	1.35±0.27^*^	1.61±0.60^*^	1.05±0.11	1.10±0.38
kaempferol-3-O-glucosyl-7-O-rhamnoside^a^^,^ ^b^	1.22±0.19	1.55±0.78	0.96±0.18	1.01±0.30
kaempferol-3-O-galactosyl-7-O-rhamnoside^b^	1.36±0.36^*^	1.48±0.59	1.14±0.28	0.97±0.31
kaempferol-3-O-glucosyl (1-2)-galactoside-7-O-rhamnoside^a^^,^ ^b^	1.09±0.28	1.54±0.68	0.96±0.20	0.73±0.29
kaempferol-3-O-glucosyl (1-2)-glucoside-7-O-rhamnoside^a^^,^ ^b^	2.53±1.08^**^	1.77±0.74^*^	0.93±0.32	0.82±0.26
*kaempferol-6-deoxyhexose^b^*	1.22±0.58	1.51±0.61	0.85±0.44	1.05±0.39
Total kaempferol (after acid hydrolysis)	1.37±0.31^*^	1.24±0.21^*^	1.07±0.30	1.31±0.53
*Quercetin-6-deoxyhexose, -hexose^b^*	20.81±9.13^**^	1.76±0.85^*^	0.99±0.65	1.08±0.51
*Quercetin-6-deoxyhexose, -hexose^b^*	15.33±9.49^**^	1.62±1.31	1.23±0.83	3.29±1.92^*^
*Quercetin-6-deoxyhexose, 6-deoxyhexose^b^*	8.78±6.24^**^	1.45±0.87	0.96±0.64	1.19±0.70
Total quercetine (after acid hydrolysis)	11.68±4.02^**^	2.63±0.50^**^	0.86±0.45	1.56±0.54^*^
*flavonol-6-deoxyhexose-hexose ^b^*	1.46±0.79	1.39±0.59	0.88±0.38	1.21±0.52
p-coumaric acid	0.33±0.16^*^	0.98±0.26	1.03±0.37	1.26±0.30
p-ferulic acid	0.81±0.52	1.31±0.74	1.16±0.51	1.13±0.48
*Simple phenylpropanoid*	0.44±0.25^**^	0.96±0.56	1.03±0.38	1.26±0.47
Vestitol	1.20±0.35	2.08±0.52^**^	1.24±0.15^*^	8.94±4.13^**^
Total tannins	1.02±0.10	1.20±0.28	1.24±0.23	1.19±0.10

The changes in the relative levels of phenolic compounds in the two genotypes as a consequence of drought or active PR are reported as the ratio between metabolite levels under control conditions (high CO_2_, normal watering) and metabolite levels under stress conditions. Total crude extracts from leaves were obtained in 50% methanol and analyzed by HPLC as described in materials and methods, with the exception of vestitol, that due to its low polarity was quantified in leaf extracts obtained in 100% methanol. The different compounds were identified in HPLC chromatograms employing DAD detector, tannins were determined spectrophotometrically. Identities of compounds were verified by

a NMR or

b *LC/ESI-MS*.

For the compounds in italics it was not possible to determine their accurate chemical structure. Significant difference between levels in mutant and WT at

* p < 0.05 or

** *p < 0.01 (n = 4) according to Student's t-test*.

*Data are the mean ± S.D. of six independent biological replicates*.

Regarding isoflavonoids, vestitol was the most abundant isoflavan phytoalexin in leaves of Lotus as also reported by Lanot and Morris (2005). Important differences were observed among WT and mutant plants regarding vestitol. Vestitol levels were increased particularly in the mutant genotype under drought, and much more (around 9-fold) under active photorespiratory conditions (Table 5). This was in very good agreement with the high induction of the genes for isoflavonoids biosynthesis observed in Ljgln2-2 mutant plants under active PR, as described above. Nevertheless, a very slight but significant increase in vestitol was also observed in the WT as a consequence of the transfer to active photorespiratory conditions.

While many of the genes for the biosynthesis of anthocyanins and tannins were modulated (Table 3), no detectable levels of anthocyanins were found in the leaves of the samples used in this study (data not shown). This confirms previous reports that failed to detect anthocyanins in L. japonicus leaves despite of a high expression of the corresponding biosynthetic gene probesets (Shimada et al., 2005). The total tannin content did not change significantly in any condition (Table 5).

## Discussion

The present work has shown the existence of important changes in phenolic metabolism as a result of GS_2_ deficiency in L. japonicus plants in response to stress. These changes were first detected from a transcriptomic analysis of the plants combined with further qRT-PCR validation, followed by phenolic metabolites profiling and characterization. Two different kinds of stress situations were examined: on the one hand, drought stress in the absence of active photorespiration, and, on the other hand, the stress produced as a result of the impairment of the photorespiratory cycle due to the lack of GS_2_, under normal watering. Transcriptomic analysis indicated the existence of a pattern of convergent responses in L. japonicus mutants lacking GS_2_ when comparing both types of stress situations, in spite of the fact that they are very different stress conditions. Therefore, the lack of GS_2_ must be associated with the common changes that were observed in both types of stress. Bioinformatics analysis indicated that the genes for the biosynthesis of phenolic compounds were over-represented among the group of genes that changed under both types of stress situations.

Phenylpropanoid and flavonoid biosynthesis were found to be generally stimulated in leaves of L. japonicus in stress, particularly as a result of GS_2_ deficiency. qRT-PCR analysis indicated that several genes for the biosynthesis of phenolic compounds were highly induced by both stress conditions, especially in the mutant. In fact, the gene probesets encoding for the enzymes that catalyze the “entry” reactions of the pathway (PAL, C4H, and 4CL) were highly induced in the mutant under both stress conditions.

Flavonols are probably the most important flavonoids participating in stress response and show a wide range of biological activities (Falcone Ferreyra et al., 2012). An increasing body of evidence is indicating that flavonols, especially those with dihydroxy-subsituted B-ring, play an antioxidant role in plant stress response (Pollastri and Tattini, 2011). In this paper it was observed that kaempferol and quercetine, the main flavonols detected in L. japonicus leaves, tended to accumulate in response to stress. In contrast, in control conditions, the levels of phenolic compounds was similar in the mutant plants than in the WT, except for some slight increase in quercetine glycosides in the mutant plants, that could be compensating for the also slight decrease observed in the mutants in some kaempferol glycosides. Flavonol accumulation may represent a defense against the increased oxidative stress produced by drought (that would affect both genotypes) or active PR (that affects mainly the Ljgln2-2 mutant). In fact, previous works have shown an increased level of oxidative stress in L. japonicus plants as a result of drought (Díaz et al., 2010) or active photorespiration in the mutant plants (Pérez-Delgado et al., 2013). Consistent with this theory, WT plants under active photorespiratory conditions did not accumulate flavonoids, with the exception of a minor increase in vestitol levels. It is important to note that active photorespiration should not mean any stress situation for the WT plants since they have a normal operation of the photorespiratory cycle, in contrast with the Ljgln2-2 mutant plants, which have an impairment of the photorespiratory cycle due to the lack of GS_2_. However, drought stress produced a stress situation that was clearly apparent both in WT and mutant plants.

Several kaempferol glycosides were identified in the L. japonicus leaf extracts used in this work. Some of these compounds have been described in previous reports like Suzuki et al. (2008), including kaempferol-3,7-di-O-rhamnoside that was the most abundant kaempferol glycoside in L. japonicus leaves under control conditions. Suzuki et al. (2008) also detected kaempferol-3-O-glucosyl-7-O-rhamnoside and kaempferol-3-O-galactosyl-7-O-rhamnoside. On the other hand, two triple glycosides of kaempferol were detected in this work and one of them, kaempferol-3-O-glucosyl (1-2)-glucoside-7-O-rhamnoside, showed the higher change in relative metabolite content under stress. This particular triple glycoside has been also identified in Arabidopsis leaves (Kachlicki et al., 2008). The other kaempferol triple glycoside detected, kaempferol-3-O-glucosyl (1-2)-galactoside-7-O-rhamnoside, did not accumulate significantly under stress conditions and, to our knowledge, was detected for the first time in plants.

We call attention now to the fact that a massive increase of quercetine and a slight increase of kaempferol compounds were detected in the WT plants under stress conditions, whereas in the mutant, the main phenolic pathway that was altered was the biosynthesis and accumulation of isoflavonoids, particularly under photorespiratory active conditions. This is in good agreement with the gene-expression data showing a strong induction of both PAL, which is the primary mediator of the flux toward the phenylpropanoid pathway under all stress conditions and of several genes encoding for enzymes of isoflavonoid pathway, mainly in the GS_2_ deficient mutant plants. The data obtained indicate that the increased flux toward the phenylpropanoid pathway is probably directed toward vestitol production in the mutant plants while in the WT this increased flux is probably aimed to increased flavonols production. In fact, accumulation of vestitol, the main isoflavonoid normally present in L. japonicus leaves (Lanot and Morris, 2005) was substantially increased in the mutant plants under the two types of stress conditions examined, most particularly under active photorespiration. Vestitol is a typical phytoalexin, mainly active in the response to pathogen attack (Shimada et al., 2000). However, there are also reports showing that, in legumes, accumulation of isoflavonoid phytoalexins and induction of their biosynthesis may also occur in different types of abiotic stresses such as UV-irradiation, drought or presence of heavy metals (Parry et al., 1994; Yamaguchi et al., 2010; Zavala et al., 2014). Interestingly, the previous work of Pérez-Delgado et al. (2013) showed that active PR caused, in the Ljgln2-2 mutant, an increased production of H_2_O_2_ and an increase in transcript levels and enzyme activity of a glycolate oxidase isoform that was positively co-expressed with gene probesets involved in the response to biotic stress. Therefore, it is possible that the stress perceived by the mutant under active PR may mimic some stages of the signal transduction pathway that is elicited by biotic stress thus stimulating the biosynthesis of isoflavonoids over the flavonols. For soybean, a strong bias toward increasing the expression of gene probesets for isoflavonoid phytoalexin synthesis was documented after infection with Pseudomonas syringae, that was concomitant with some down regulation of gene probesets involved in the synthesis of other groups of flavonoids comprising flavonols, anthocyanins, and tannins (Zabala et al., 2006). Moreover, a high induction of several gene probesets for isoflavonoids biosynthesis was reported also by Shimada et al. (2007) and Shelton et al. (2012) when isoflavonoids production was elicited in L. japonicus leaves using reduced glutathione, a treatment that induces mainly gene probesets associated with the response to biotic challenges like the ones for phytoalexin production (Shimada et al., 2000; Foyer and Noctor, 2005; Shelton et al., 2012).

In summary, the results shown in the present paper indicate that the presence or absence of GS_2_ produce important differences in the different pathways for phenolics biosynthesis in L. japonicus in response to stress. While the presence of GS_2_ results in a high increase in flavonols (quercetine and/or kaempherol), as detected in the WT, the absence of GS_2_ results in a high increase in isoflavonoids (vestitol) in the mutant plants. Thus, it can be concluded a clear implication of GS_2_ in phenolic metabolism and in the different stress responses L. japonicus plants. The fact that isoflavonoid metabolism is particularly important in legume plants, in difference with other plants species such as Arabidopsis, makes the results obtained in L. japonicus of special interest. It is possible that GS_2_ may be connected with some type of regulatory network related to phenolic metabolism in L. japonicus plants. Previous works have established the crucial role of GS_2_ in the C/N balance of L. japonicus plants (García-Calderón et al., 2012; Betti et al., 2014). On the other hand, different lines of evidence suggested that the response of Ljgln2-2 mutants to either drought stress or active photorespiration involve oxidative stress. The increase in thiobarbituric acid-reactive species observed in the mutant under drought conditions was significantly higher than in the WT (Díaz et al., 2010), indicating higher levels of oxidative stress in this genotype. In addition, the reactivation of the photorespiratory cycle was paralleled by accumulation of H_2_O_2_ exclusively in the mutant (Pérez-Delgado et al., 2013). Different kinds of abiotic stress are known to induce the accumulation of reactive oxygen species, and as a consequence of that, trigger several antioxidant defenses in the plant (Nakabayashi and Saito, 2015). Moreover, reactive oxygen species are also part of signaling cascades that modulate several signal transduction pathways related to abiotic stress (Jaspers and Kangasjärvi, 2010). Further work would be still required to analyze the regulatory networks and transcription factors that may regulate the different branches of the flavonoid pathway, a very actual and interesting topic that needs further investigation especially in legumes.

The results presented here constitute a substantial advance in the study of the response of the model legume L. japonicus to different stress situations, making use for this purpose of Ljgln2-2 photorespiratory mutants deficient in GS_2_. Transcriptomic analysis and qRT-PCR studies revealed the stimulation of the expression of several genes for the biosynthesis of phenolic compounds under stress conditions, particularly in the mutant plants. A differential response among WT and mutant plants was observed regarding the biosynthesis and accumulation of different branches of flavonoids such as flavonols (quercetin/kaempferol) or isoflavonoids (vestitol). In addition, accumulation of several flavonol glycosides, some of them described for the first time in here, was also observed. Therefore, the results obtained constitute very novel and interesting findings which point out a crucial relevance of GS_2_ in relation to phenolic metabolism and stress responses of L. japonicus plants, thus providing a very nice example of functions beyond primary metabolism of the amino acids of the glutamate family, which is the research topic that was analyzed.

## Author contributions

The experiments were conceived and designed by PP, JV, MR, AM, and MB. The experiments were carried out by MG, TP, AM, CP, MV, and AE. The data were analyzed by PP, MR, CP, MV, and MB. The paper was written by PP, AM, and MB.

### Conflict of interest statement

The authors declare that the research was conducted in the absence of any commercial or financial relationships that could be construed as a potential conflict of interest.

## Supplementary material

Supplemental Figure S1 **Experimental design used in this work**. WT and *Ljgln2-2* mutant plants were grown under high CO_2_ (0.7% v/v) conditions and watered with Hornum medium. After 35 days leaf samples were harvested from both genotypes, constituting the control condition for both experiments. For drought treatment, plants were kept under high CO_2_ conditions and watering was withheld for 4 days, after which leaf samples were harvested. For active PR treatment, plants were transferred for 2 days to a normal CO_2_ (0.04% v/v) atmosphere and leaf samples were harvested. More details in materials and methods.

Supplemental Figure S2 **Mapman overview of general metabolism of the gene probesets that changed under both types of stress situations in WT plants**. Since the change in gene expression levels for these gene probesets was different in the response to drought and active PR, an arbitrary fold-change of 2 was imposed. More details about the graphical representation of genes in the context of metabolic pathways used by the MapMan software can be found in the legend of Figure 1.

Supplemental Figure S3 **Relationship of the fold-change in gene expression levels produced by drought or active photorespiratory conditions for the different gene probesets modulated exclusively in the ***Ljgln2-2*** mutant. (A)** Graphical representation of the log_2_ of the fold-change for all the 2173 gene probesets that were modulated exclusively in the mutant under drought or active PR conditions. **(B)** Gene probesets that changed in the same direction (commonly induced or repressed) under drought or active photorespiratory conditions. The linear regression analysis carried out gave a r^2^ value of 0.80 and a slope of 0.60. **(C)** Gene probesets that changed in opposite directions in response to drought and active PR. The linear regression analysis carried out gave a r^2^ value of 0.77 and a slope of −0.52.

Supplemental Figure S4 **Comparison of microarray and qRT-PCR data for genes involved in the biosynthesis of phenolic compounds**. The points plotted in this graph represent the log_2_ of the fold-change for different gene probesets involved in the biosynthesis of phenolic compounds that were significantly modulated according to both techniques. The data points plotted are from both WT and *Ljgln2-2* genotypes and both drought or active photorespiratory conditions. Linear regression analysis of microarray data versus qRT-PCR data gave a regression coefficient of *r*^2^ = 0.92 and a slope of 0.81.

Supplemental Figure S5 **Data of the NMR spectra used for the identification of the kaempferol glycosides detected in this work**.

Supplemental Figure S6 **HPLC elution profiles of flavonoids from ***L. japonicus*** leaves of (A) WT plants under high CO_2_ conditions and (B) WT plants after 2 days under normal CO_2_ conditions**. The compounds were detected according to their absorbance at 280 nm. Correspondence between peak numbers and metabolites as described in the legend of Figure 4. The chromatograms shown here are for only one of the six different biological replicates used in this work. For the relative quantification of metabolite levels presented in Table 5 the mean of all the six replicates was used.

Supplemental Figure S7 **HPLC elution profiles of flavonoids from ***L. japonicus*** leaves of (A) ***Ljgln2-2*** plants under normal watering conditions and (B) ***Ljgln2-2*** plants under drought stress conditions**. The compounds were detected according to their absorbance at 280 nm. Correspondence between peak numbers and metabolites as described in the legend of Figure 4. The chromatograms shown here are for only one of the six different biological replicates used in this work. For the relative quantification of metabolite levels presented in Table 5 the mean of all the six replicates was used.

Supplemental Figure S8 **HPLC elution profiles of flavonoids from ***L. japonicus*** leaves of (A) ***Ljgln2-2*** plants under high CO_2_ conditions and (B) ***Ljgln2-2*** plants after 2 days under normal CO_2_ conditions**. The compounds were detected according to their absorbance at 280 nm. Correspondence between peak numbers and metabolites as described in the legend of Figure 4. The chromatograms shown here are for only one of the six different biological replicates used in this work. For the relative quantification of metabolite levels presented in Table 5 the mean of all the six replicates was used.

Supplemental Table S1 **Oligonucleotides used for qRT-PCR analysis**. The gene copy amplified by each oligonucleotide pair is indicated. In some cases, more than one gene copy was amplified by the oligonucleotides due the high homology among different members of the gene family; in this case the different gene copies recognized are specified in the column “Gene(s) amplified.” For an exhaustive list of the gene copies encoding for each of the enzymes considered in this work see Supplemental Table S4.

Supplemental Table S2 **List of the gene probesets that changed under the two types of stress situations in WT and mutant plants**. The list of the 187 gene probesets that changed under both types of stress treatments in the WT plants and of the 2173 that changed under both types of stress treatments in the mutant are presented together with a description of the product of each gene.

Supplemental Table S3 **Top 20 genes that were induced bydrought or active photorespiration in the ***Ljgln2-2*** mutant**. The highlighted genes are those that were in common among the the list of 20 most induced genes that were observed in the drought stress or active PR treatments.

Supplemental Table S4 **List of the available gene sequences for the biosynthesis of phenolic compounds found in the current release of the ***L. japonicus*** genome**. Data mining was carried out in the 2.5 release of *L. japonicus* genome at the Kazusa institute database (http://www.kazusa.or.jp/lotus/). The gene names for the members of each gene family were defined in this work or, when available, taken from the bibliography.

Supplemental Table S5 **LC/ESI-MS data of flavonol glycosides identified in 50% methanol extracts from ***L. japonicus*** leaves**. Correspondence between peak numbers and metabolites as described in the legend of Figure 5.

## References

[B1] Bauer D. Biehler K. Fock H. Carrayol E. Hirel B. Miggie A. (1997). A role for cytosolic glutamine synthetase in the remobilization of leaf nitrogen during water stress in tomato. Physiol. Plant. 99, 241–247. 10.1111/j.1399-3054.1997.tb05408.x

[B2] Betti M. García-Calderón M. Pérez-Delgado C. M. Credali A. Estivill G. Galván F.. (2012). Glutamine synthetase in legumes: recent advances in enzyme structure and functional genomics. Int. J. Mol. Sci. 13, 7994–8024. 10.3390/ijms13077994 22942686 PMC3430217

[B3] Betti M. García-Calderón M. Pérez-Delgado C. M. Credali A. Pal'ove-Balang P. Estivill G.. (2014). Reassimilation of ammonium in Lotus japonicus. J. Exp. Bot. 65, 5557–5566. 10.1093/jxb/eru260 24948681

[B4] Brugière N. Dubois F. Limami A. M. Lelandais M. Roux Y. Sangwan R. S.. (1999). Glutamine synthetase in the phloem plays a major role in controlling proline production. Plant Cell 11, 1995–2011. 10.1105/tpc.11.10.1995 10521528 PMC144111

[B5] Cheynier V. Comte G. Davies K. M. Lattanzio V. Martens S. (2013). Plant phenolics: recent advances on their biosynthesis, genetics, and ecophysiology. Plant Physiol. Biochem. 72, 1–20. 10.1016/j.plaphy.2013.05.009 23774057

[B6] Czechowski T. Bari R. P. Stitt M. Scheible W. R. Udvardi M. K. (2004). Real-time RT-PCR profiling of over 1400 Arabidopsis transcription factors: unprecedented sensitivity reveals novel root- and shoot-specific genes. Plant J. 38, 366–379. 10.1111/j.1365-313X.2004.02051.x 15078338

[B7] Davies K. M. Schwinn K. E. (2006). Molecular biology and biotechnology of flavonoid biosynthesis, in Flavonoids: Chemistry, Biochemistry and Applications, ed Andersen Ø. M. Markham K. R. (Boca Ratón, FL: Taylor & Francis Group), 143–218.

[B8] Díaz P. Betti M. Sánchez D. H. Udvardi M. K, Monza, J. Márquez A. J. (2010). Deficiency in plastidic glutamine synthetase alters proline metabolism and transcriptomic response in *Lotus japonicus* under drought stress. New Phytol. 188, 1001–1013. 10.1111/j.1469-8137.2010.03440.x 20796214

[B9] Falcone Ferreyra M. L. Rius S. P. Casati P. (2012). Flavonoids: biosynthesis, biological functions, and biotechnological applications. Front. Plant Sci. 3:222. 10.3389/fpls.2012.00222 23060891 PMC3460232

[B10] Foyer C. H. Noctor G. (2005). Oxidant and antioxidant signalling in plants: a re-evaluation of the concept of oxidative stress in a physiological context. Plant Cell Environ. 28, 1056–1071. 10.1111/j.1365-3040.2005.01327.x

[B11] García-Calderón M. Chiurazzi M. Espuny M. R. Márquez A. J. (2012). Photorespiratory metabolism and nodule function: behavior of Lotus japonicus mutants deficient in plastid glutamine synthetase. Mol. Plant-Microbe Interact. 25, 211–219. 10.1094/MPMI-07-11-0200 22007601

[B12] Goffard N. Weiller G. (2007). PathExpress: a web-based tool to identify relevant pathways in gene expression data. Nucleic Acids Res. 35, W176–W818. 10.1093/nar/gkm261 17586825 PMC1933187

[B13] Handberg K. Stougaard J. (1992). *Lotus japonicus*, an autogamous, diploid legume species for classical and molecular genetics. Plant J. 2, 487–496. 10.1111/j.1365-313X.1992.00487.x

[B14] Heigl D. Franz G. (2003). Stability testing on typical flavonoid containing herbal drugs. Parmazie 58, 881–885. 14703966

[B15] Hoshida H. Tanaka Y. Hibino T. Hayashi Y. Tanaka A. Takabe T.. (2000). Enhanced tolerance to salt stress in transgenic rice that overexpress chloroplast glutamine synthetase. Plant Mol. Biol. 43, 103–111. 10.1023/A:1006408712416 10949377

[B16] Jaspers P. Kangasjärvi J. (2010). Reactive oxygen species in abiotic stress signalling. Physiol. Plant. 138, 405–413. 10.1111/j.1399-3054.2009.01321.x 20028478

[B17] Kachlicki P. Einhorn J. Muth D. Kerhoas L. Stobiecki M. (2008). Evaluation of glycosylation and malonylation patterns in flavonoid glycosides during LC/MS/MS metabolite profiling. J. Mass Spectrom. 43, 572–586. 10.1002/jms.1344 18074333

[B18] Kozaki A. Takeba G. (1996). Photorespiration protects C3 plants from photooxidation. Nature 384, 557–560. 10.1038/384557a0

[B19] Lanot A. Morris P. (2005). Elicitation of isoflavan phytoalexins, in Lotus japonicus Handbook, ed Márquez A. J. (Dordrecht: Springer), 355–361. 10.1007/1-4020-3735-X_35

[B20] Nakabayashi R. Yonekura-Sakakibara K. Urano K. Suzuki M. Yamada Y. Nishizawa T.. (2014). Enhancement of oxidative and drought tolerance in Arabidopsis by overaccumulation of antioxidant flavonoids. Plant J. 77, 367–379. 10.1111/tpj.12388 24274116 PMC4282528

[B21] Nakabayashi R. Saito K. (2015). Integrated metabolomics for abiotic stress responses in plants. Curr. Opin. Plant Biol. 24, 10–16. 10.1016/j.pbi.2015.01.003 25618839

[B22] Orea A. Pajuelo P. Pajuelo E. Quidiello C. Romero J. M. Márquez A. J. (2002). Isolation of photorespiratory mutants from *Lotus japonicus* deficient in glutamine synthetase. Physiol. Plant. 115, 352–361. 10.1034/j.1399-3054.2002.1150304.x 12081527

[B23] Pal'ove-Balang P. Betti M. Díaz P. Pérez-Delgado C. M. García-Calderón M. Monza J. (2014). Abiotic stress in Lotus: aluminium and drought, in Molecular Approaches in Plant Abiotic Stress, eds Gaur R. K. Sharma P. (Boca Raton, FL: CRC Press), 284–303.

[B24] Parry A. D. Tiller S. A. Edwards R. (1994). The effects of heavy metals and root immersion on isoflavonoid metabolism in alfalfa (*Medicago sativa*). Plant Physiol. 106, 195–202. 12232319 10.1104/pp.106.1.195 PMC159516

[B25] Pérez-Delgado C. M. García-Calderón M. Sánchez D. H. Udvardi M. K. Kopka J. Márquez A. J.. (2013). Transcriptomic and metabolic changes associated with photorespiratory ammonium accumulation in the model legume *Lotus japonicus*. Plant Physiol. 162, 1834–1848. 10.1104/pp.113.217216 23743713 PMC3729765

[B26] Pollastri S. Tattini M. (2011). Flavonols: old compounds for old roles. Ann. Bot. 108, 1225–1233. 10.1093/aob/mcr234 21880658 PMC3197460

[B27] Sánchez D. H. Lippold F. Redestig H. Hannah M. A. Erban A. Krämer U.. (2008). Integrative functional genomics of salt acclimatation in the model legume Lotus japonicus. Plant J. 53, 973–987. 10.1111/j.1365-313X.2007.03381.x 18047558

[B28] Saito K. Yonekura-Sakakibara K. Nakabayashi R. Higashi Y. Yamazaki M. Tohge T.. (2013). The flavonoid biosynthetic pathway in Arabidopsis: structural and genetic diversity. Plant Physiol. Biochem. 72, 21–34. 10.1016/j.plaphy.2013.02.001 23473981

[B29] Shelton D. Stranne M. Mikkelsen L. Pakseresht N. Welham T. Hiraka H.. (2012). Transcription Factors of Lotus: regulation of isoflavonoid biosynthesis requires coordinated changes in transcription factor activity. Plant Physiol. 159, 531–547. 10.1104/pp.112.194753 22529285 PMC3375922

[B30] Shimada N. Akashi T. Aoki T. Ayabe S.-I. (2000). Induction of isoflavonoids pathway in the model legume *Lotus japonicus*: molecular characterization of enzymes involved in phytoalexin biosynthesis. Plant Sci. 160, 37–47. 10.1016/S0168-9452(00)00355-1 11164575

[B31] Shimada N. Aoki T. Sato S. Nakamura Y. Tabata S. Ayabe S.-I. (2003). A cluster of genes encodes the two types of chalcone isomerase involved in the biosynthesis of general flavonoids and legume-specific 5-deoxy(iso)flavonoids in *Lotus japonicus*. Plant Physiol. 131, 941–951. 10.1104/pp.004820 12644647 PMC166860

[B32] Shimada N. Sasaki R. Sato S. Kaneko T. Tabata S. Aoki T.. (2005). A comprehensive analysis of six dihydroflavonol 4-reductases encoded by a gene cluster of the *Lotus japonicus* genome. J. Exp. Bot. 419, 2573–2585. 10.1093/jxb/eri251 16087700

[B33] Shimada N. Sato S. Akashi T. Nakamura Y. Tabata S. Ayabe S.-I.. (2007). Genome-wide analyses of the structural gene families involved in the legume-specific 5-deoxyisoflavonoid biosynthesis of *Lotus japonicus*. DNA Res. 14, 25–36. 10.1093/dnares/dsm004 17452423 PMC2779890

[B34] Suzuki H. Sasaki R. Ogata Y. Nakamura Y. Sakurai N. Kitajima M.. (2008). Metabolic profiling of flavonoids in *Lotus japonicus* using liquid chromatography Fourier transform ion cyclotron resonance mass spectrometry. Phytochemistry 69, 99–111. 10.1016/j.phytochem.2007.06.017 17669449

[B35] Usadel B. Nagel A. Thimm O. Redestig H. Blaesing O. E. Palacios-Rojas N.. (2005). Extension of the visualization tool MapMan to allow statistical analysis of arrays, display of corresponding genes, and comparison with known responses. Plant Physiol. 138, 1195–1204. 10.1104/pp.105.060459 16009995 PMC1176394

[B36] Winkel-Shirley B. (2002). Biosynthesis of flavonoids and effect of stress. Curr. Opin. Plant Biol. 5, 218–223. 10.1016/S1369-5266(02)00256-X 11960739

[B37] Yamaguchi M. Valliyodan B. Zhang J. Lenoble M. E. Yu O. Rogers E. E.. (2010). Regulation of growth response to water stress in the soybean primary root. I. Proteomic analysis reveals region-specific regulation of phenylpropanoid metabolism and control of free iron in the elongation zone. Plant Cell Environ. 33, 223–243. 10.1111/j.1365-3040.2009.02073.x 19906149

[B38] Zabala G. Zou J. Tuteja J. Gonzalez D. O. Clough S. J. Vodkin L. O. (2006). Transcriptome changes in the phenylpropanoid pathway of *Glycine max* in response to *Pseudomonas syringae* infection. BMC Plant Biol. 6:26. 10.1186/1471-2229-6-26 17083738 PMC1636052

[B39] Zavala J. A. Mazza C. A. Dillon F. M. Chludil H. D. Ballaré C. L. (2014). Soybean resistance to stink bugs (*Nezara viridula* and *Piezodorus guildinii*) increases with exposure to solar UV-B radiation and correlates with isoflavonoids content in pods under field conditions. Plant Cell Environ. 38, 920–928. 10.1111/pce.12368 24811566

[B40] Zhang X. Liu C.-J. (2015). Multifaceted regulations of gateway enzyme phenylalanine-ammonia lyase in the biosynthesis of phenylpropanoids. Mol. Plant 8, 17–27. 10.1016/j.molp.2014.11.001 25578269

