# Supplementary material for: Modulation of phenolic metabolism under stress conditions in a Lotus japonicus mutant lacking plastidic glutamine synthetase
Source: Front Plant Sci. 2015 Sep 25;6:760. doi: 10.3389/fpls.2015.00760 (PMC4585329; doi:10.3389/fpls.2015.00760)
Supplement: Supplemental Figure S1 — Experimental design used in this work. WT and Ljgln2-2 mutant plants were grown under high CO2 (0.7% v/v) conditions and watered with Hornum medium. After 35 days leaf samples were harvested from both genotypes, constituting the control condition for both experiments. For drought treatment, plants were kept under high CO2 conditions and watering was withheld for 4 days, after which leaf samples were harvested. For active PR treatment, plants were transferred for 2 days to a normal CO2 (0.04% v/v) atmosphere and leaf samples were harvested. More details in materials and methods. [file Presentation1.PPTX]

## Slide 1
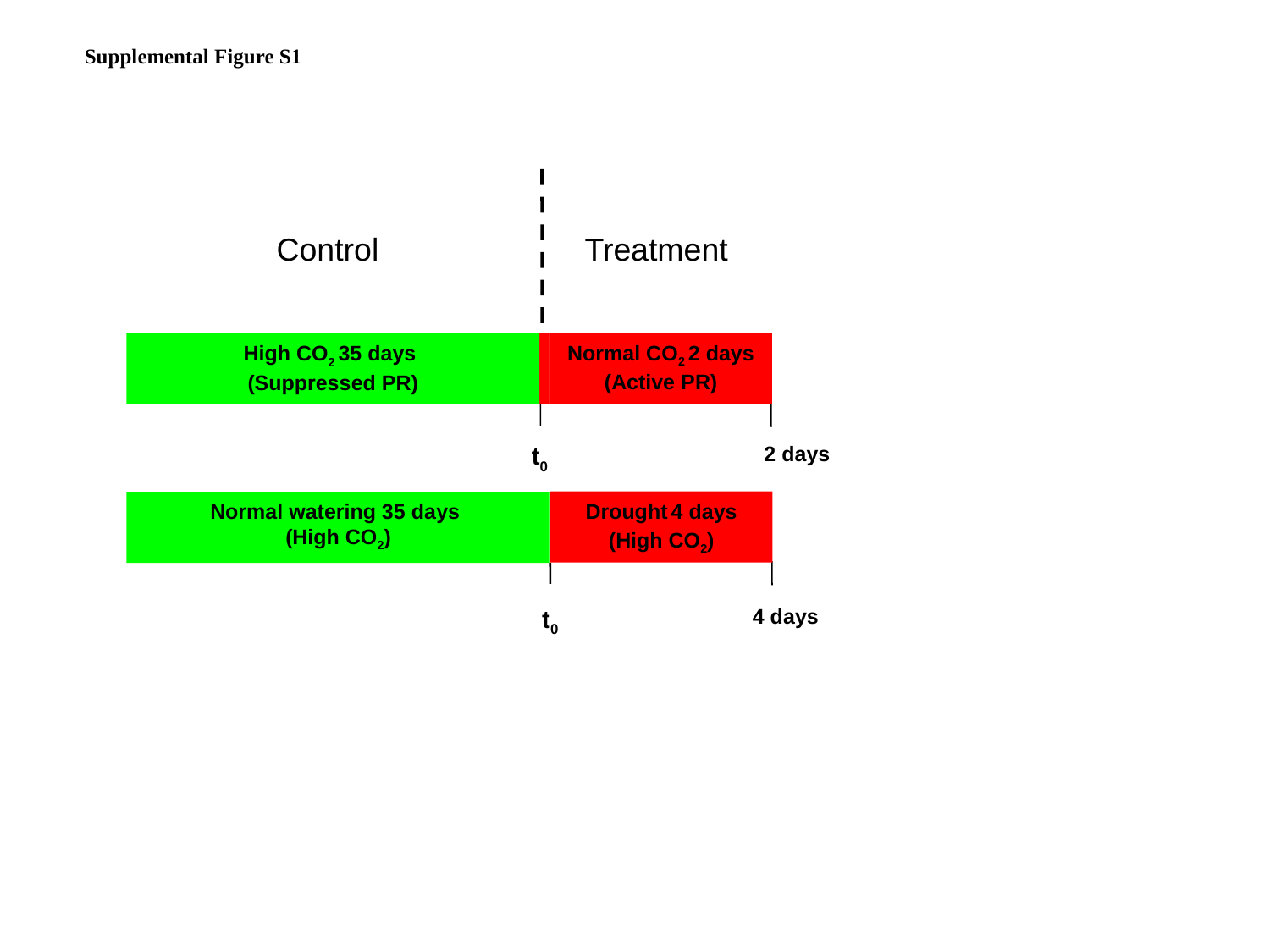

Supplemental Figure S1
Control
Treatment
High CO2 35 days
(Suppressed PR)
Normal CO2 2 days
(Active PR)
High CO2 35 days (Suppressed PR)
t0
2 days
Drought 4 days
(High CO2)
Normal watering 35 days
(High CO2)
t0
4 days
