# Supplementary material for: Modulation of phenolic metabolism under stress conditions in a Lotus japonicus mutant lacking plastidic glutamine synthetase
Source: Front Plant Sci. 2015 Sep 25;6:760. doi: 10.3389/fpls.2015.00760 (PMC4585329; doi:10.3389/fpls.2015.00760)
Supplement: Supplemental Figure S2 — Mapman overview of general metabolism of the gene probesets that changed under both types of stress situations in WT plants. Since the change in gene expression levels for these gene probesets was different in the response to drought and active PR, an arbitrary fold-change of 2 was imposed. More details about the graphical representation of genes in the context of metabolic pathways used by the MapMan software can be found in the legend of Figure 1. [file Presentation2.PPTX]

## Slide 1
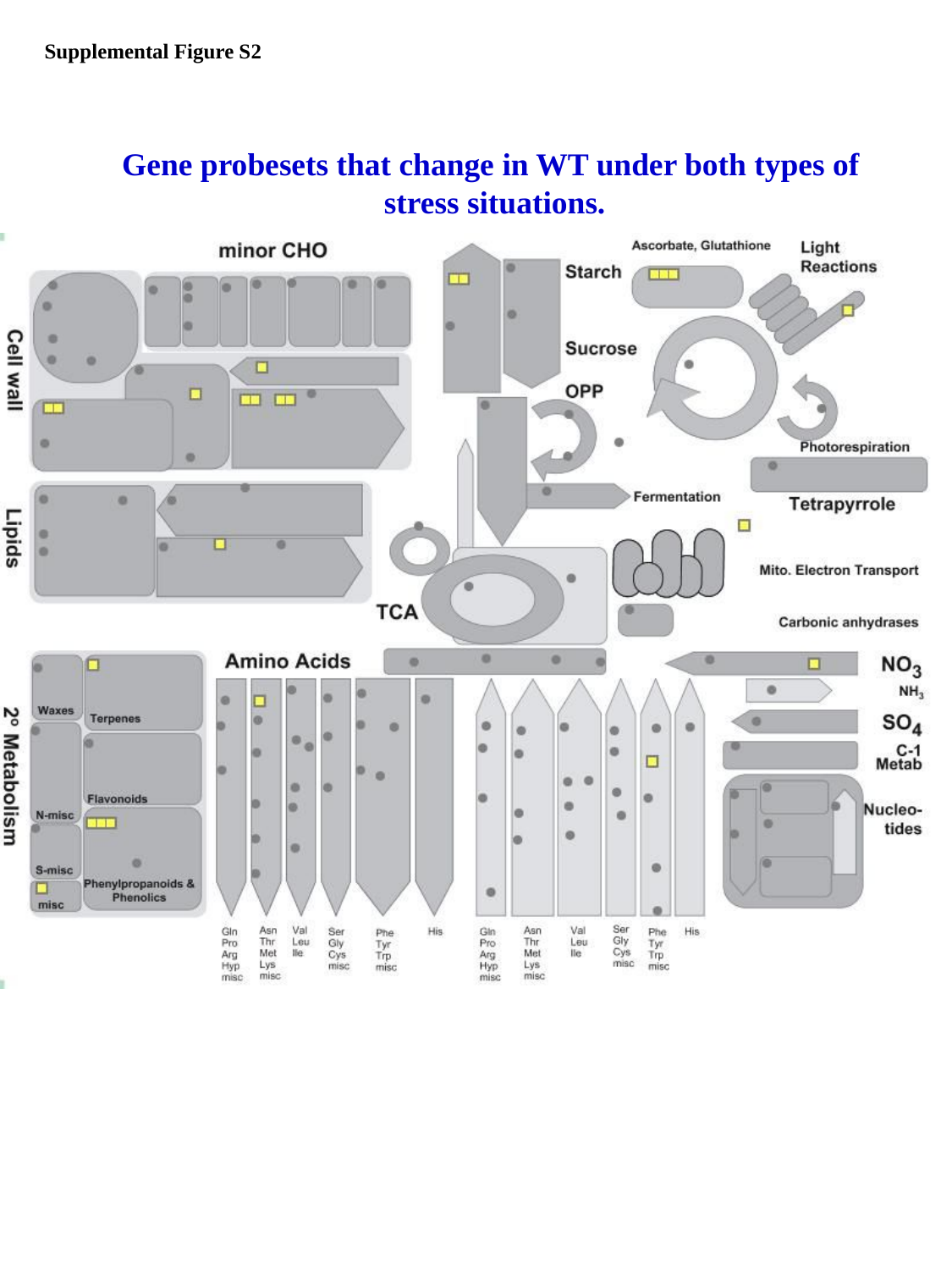

Supplemental Figure S2
Gene probesets that change in WT under both types of
stress situations.
