# Supplementary figures and images for: Modulation of phenolic metabolism under stress conditions in a Lotus japonicus mutant lacking plastidic glutamine synthetase
Source: Front Plant Sci. 2015 Sep 25;6:760. doi: 10.3389/fpls.2015.00760 (PMC4585329; doi:10.3389/fpls.2015.00760)

## Slide 1
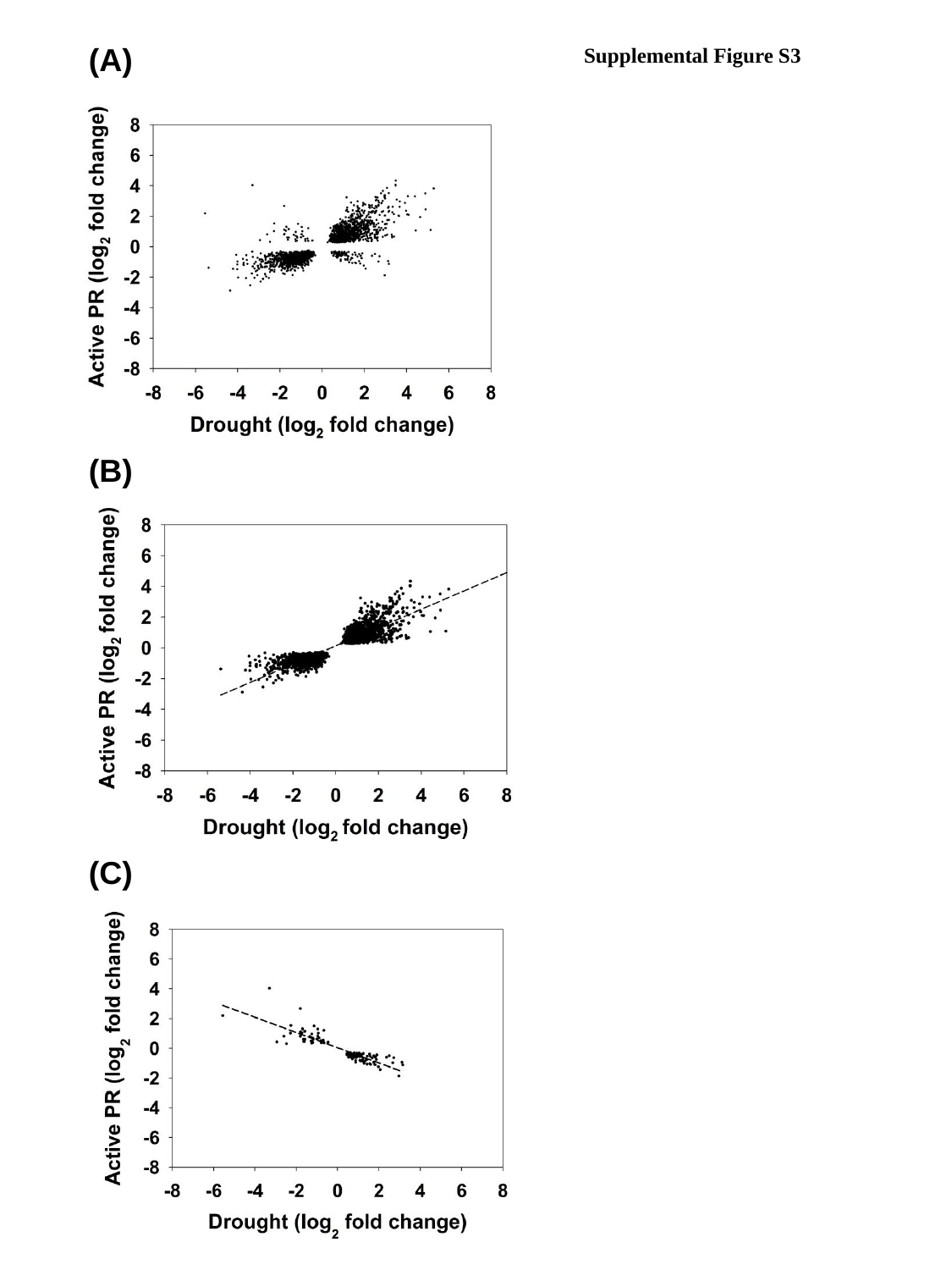

(A)
Supplemental Figure S3
(B)
(C)

Supplement: Supplemental Figure S3 — Relationship of the fold-change in gene expression levels produced by drought or active photorespiratory conditions for the different gene probesets modulated exclusively in the Ljgln2-2 mutant. (A) Graphical representation of the log2 of the fold-change for all the 2173 gene probesets that were modulated exclusively in the mutant under drought or active PR conditions. (B) Gene probesets that changed in the same direction (commonly induced or repressed) under drought or active photorespiratory conditions. The linear regression analysis carried out gave a r2 value of 0.80 and a slope of 0.60. (C) Gene probesets that changed in opposite directions in response to drought and active PR. The linear regression analysis carried out gave a r2 value of 0.77 and a slope of −0.52. [file Presentation3.PPTX]

## Slide 1
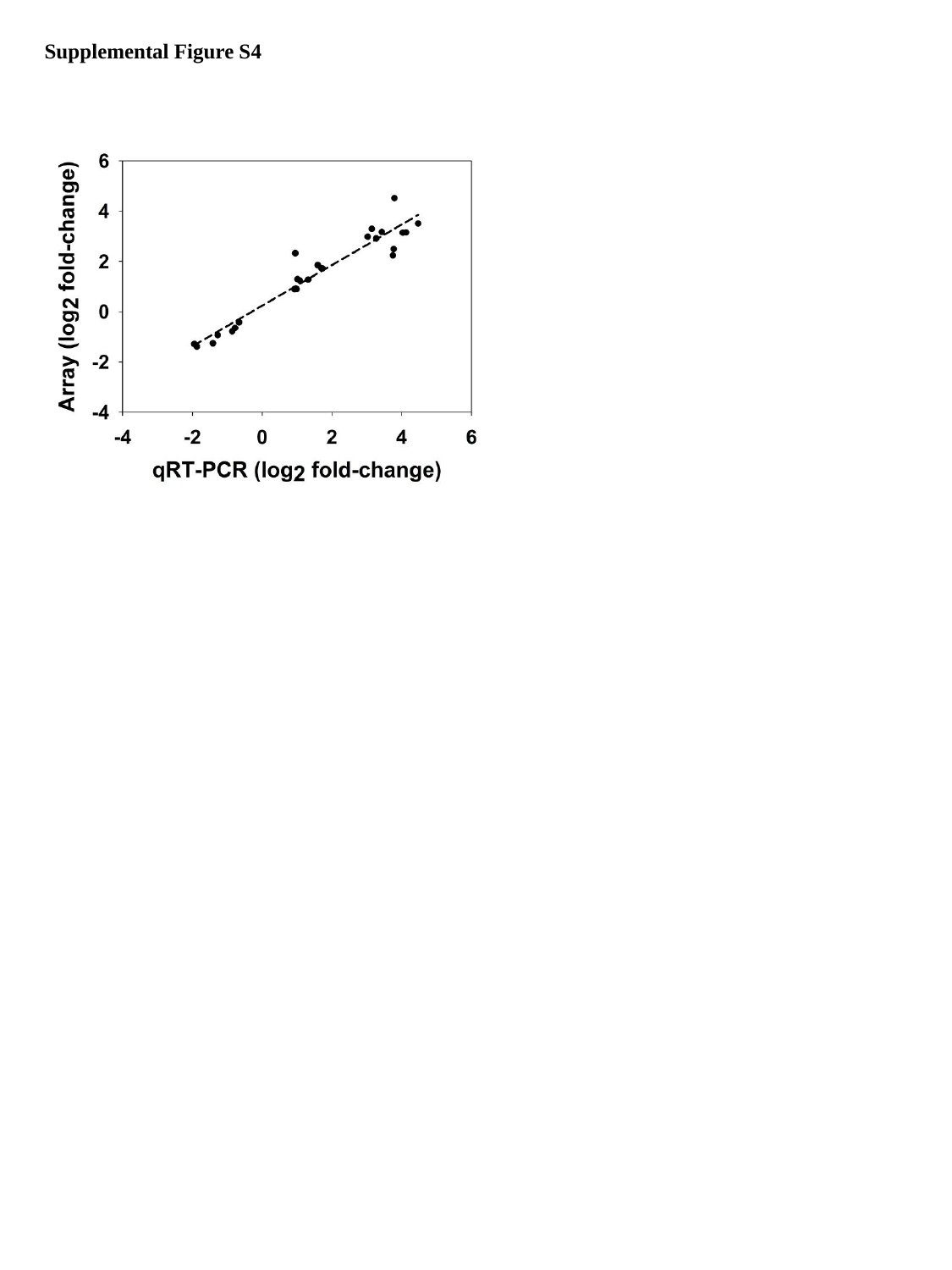

Supplemental Figure S4

Supplement: Supplemental Figure S4 — Comparison of microarray and qRT-PCR data for genes involved in the biosynthesis of phenolic compounds. The points plotted in this graph represent the log2 of the fold-change for different gene probesets involved in the biosynthesis of phenolic compounds that were significantly modulated according to both techniques. The data points plotted are from both WT and Ljgln2-2 genotypes and both drought or active photorespiratory conditions. Linear regression analysis of microarray data versus qRT-PCR data gave a regression coefficient of r2 = 0.92 and a slope of 0.81. [file Presentation4.PPTX]

## Slide 1
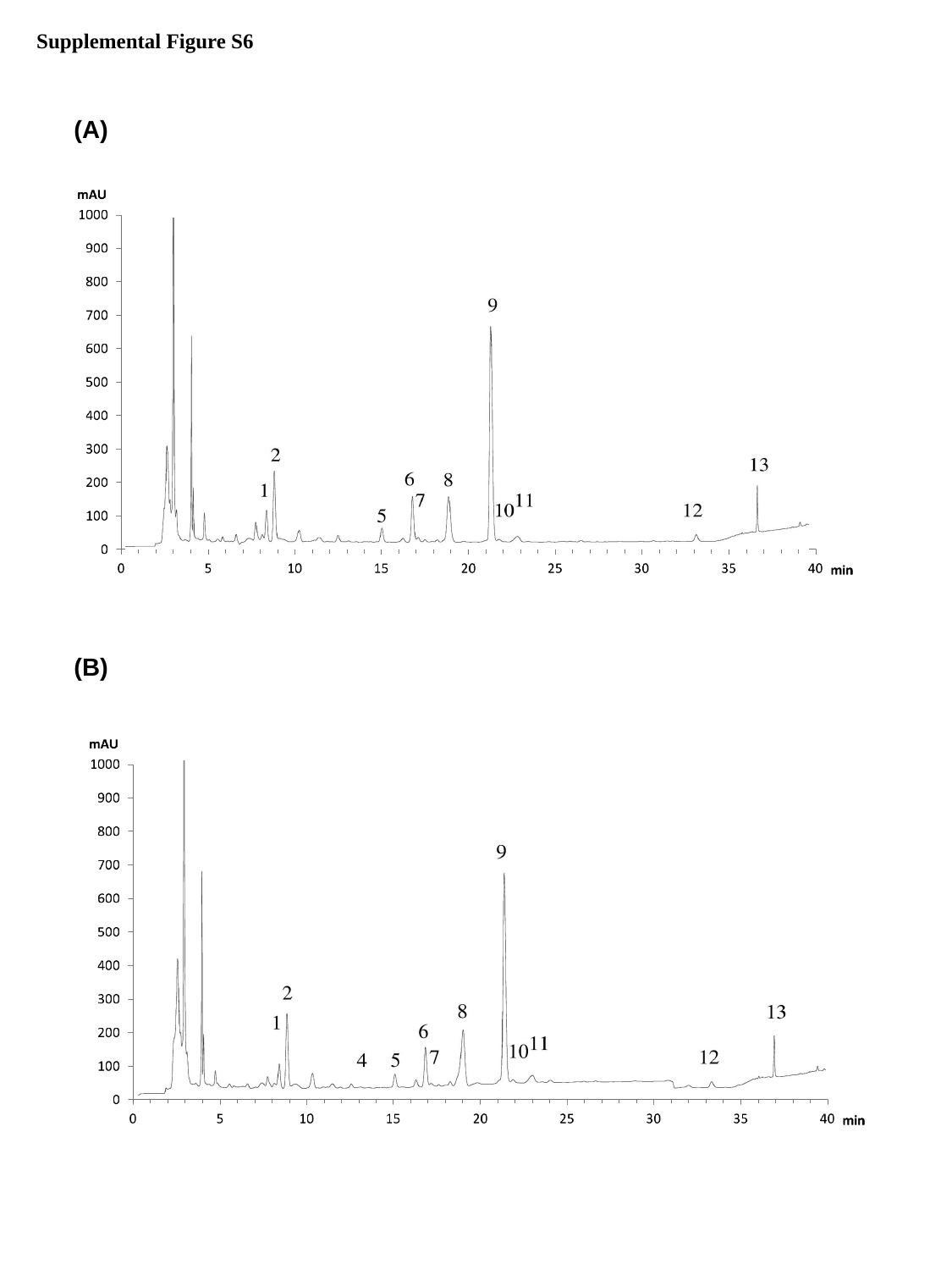

Supplemental Figure S6
(A)
(B)

Supplement: Supplemental Figure S6 — HPLC elution profiles of flavonoids from L. japonicus leaves of (A) WT plants under high CO2 conditions and (B) WT plants after 2 days under normal CO2 conditions. The compounds were detected according to their absorbance at 280 nm. Correspondence between peak numbers and metabolites as described in the legend of Figure 4. The chromatograms shown here are for only one of the six different biological replicates used in this work. For the relative quantification of metabolite levels presented in Table 5 the mean of all the six replicates was used. [file Presentation6.PPTX]

## Slide 1
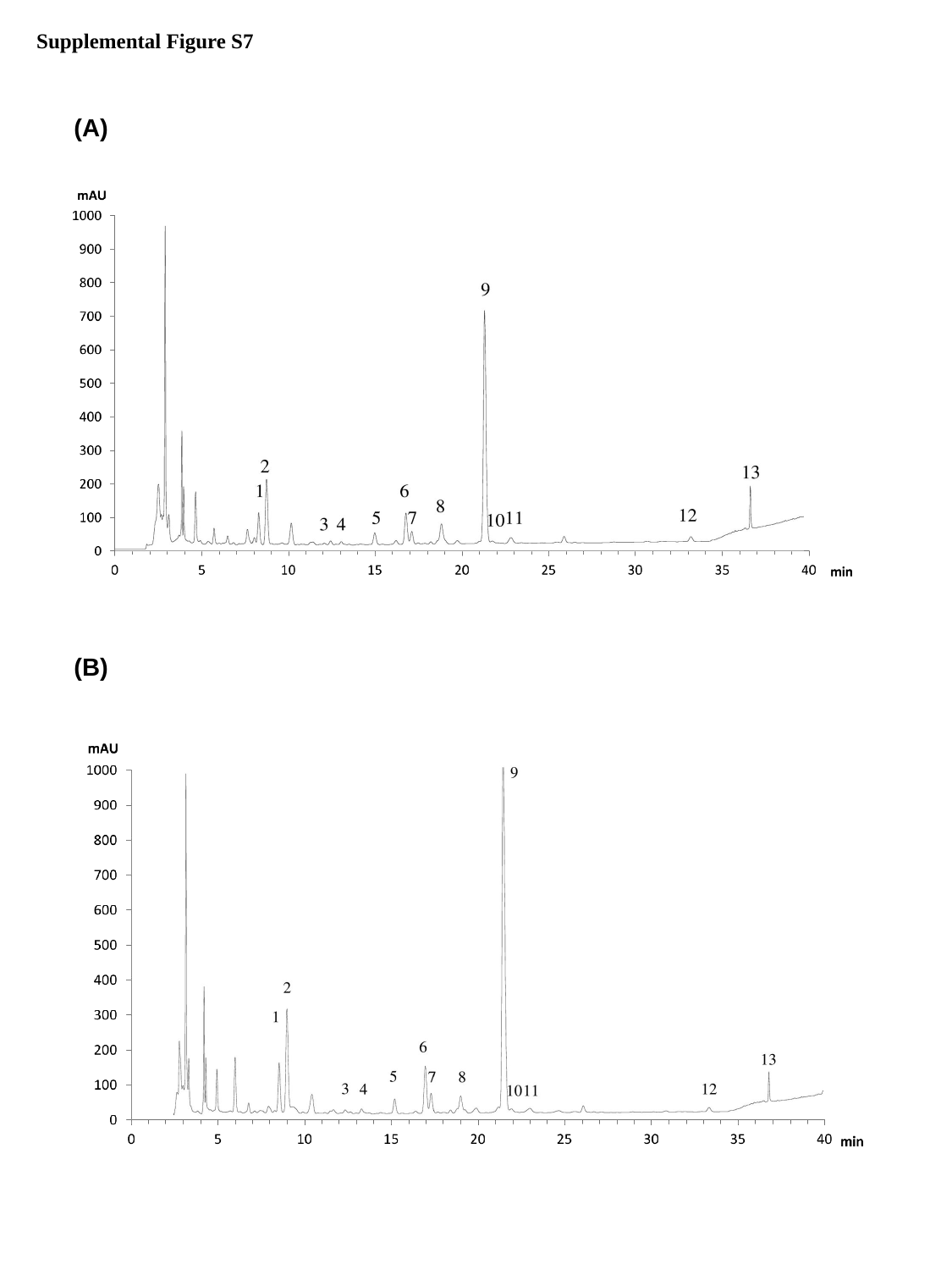

Supplemental Figure S7
(A)
(B)

Supplement: Supplemental Figure S7 — HPLC elution profiles of flavonoids from L. japonicus leaves of (A) Ljgln2-2 plants under normal watering conditions and (B) Ljgln2-2 plants under drought stress conditions. The compounds were detected according to their absorbance at 280 nm. Correspondence between peak numbers and metabolites as described in the legend of Figure 4. The chromatograms shown here are for only one of the six different biological replicates used in this work. For the relative quantification of metabolite levels presented in Table 5 the mean of all the six replicates was used. [file Presentation7.PPTX]

## Slide 1
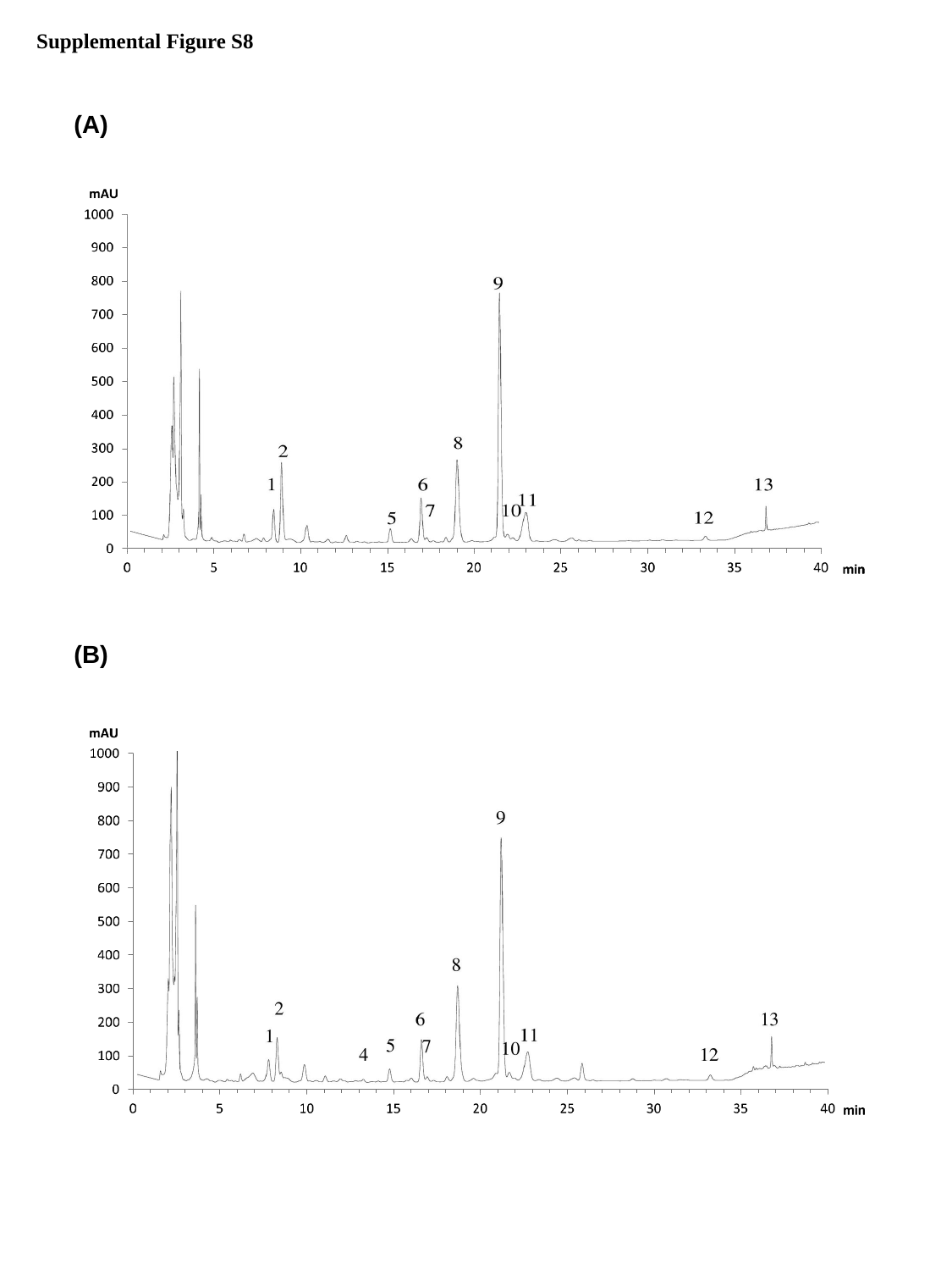

Supplemental Figure S8
(A)
(B)

Supplement: Supplemental Figure S8 — HPLC elution profiles of flavonoids from L. japonicus leaves of (A) Ljgln2-2 plants under high CO2 conditions and (B) Ljgln2-2 plants after 2 days under normal CO2 conditions. The compounds were detected according to their absorbance at 280 nm. Correspondence between peak numbers and metabolites as described in the legend of Figure 4. The chromatograms shown here are for only one of the six different biological replicates used in this work. For the relative quantification of metabolite levels presented in Table 5 the mean of all the six replicates was used. [file Presentation8.PPTX]
