# Supplementary material for: Modulation of phenolic metabolism under stress conditions in a Lotus japonicus mutant lacking plastidic glutamine synthetase
Source: Front Plant Sci. 2015 Sep 25;6:760. doi: 10.3389/fpls.2015.00760 (PMC4585329; doi:10.3389/fpls.2015.00760)
Supplement: Supplemental Figure S5 — Data of the NMR spectra used for the identification of the kaempferol glycosides detected in this work. [file Presentation5.PDF]

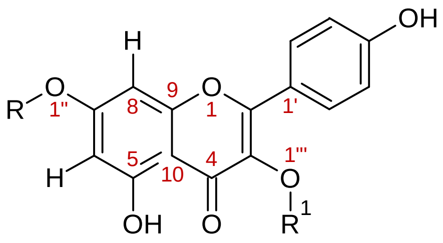

Atom numbering.

The ^{1}H-NMR spectrum of compound **1** displayed the characteristic signals of the kaempferol nucleus: two doublets at δ_H 6.74 and 6.47 ppm (*J* 2.1 Hz), assigned to the H-8 and H-6 protons, respectively, and a pair of A_2B_2 aromatic system protons at δ_H 7.80 and 6.94 ppm (*J* 8.4 Hz), assigned to H-2',6' and H-3',5' respectively. In the NMR spectrum two doublets, at δ 1.26 (d, *J* 6.0 Hz) and 0.93 (d, *J* 5.4 Hz), indicated the presence of two sets of rhamnose methyl protons and the signals for the anomeric protons appeared at δ 5.56 (d, *J* 1.8 Hz) and 5.56 (d, *J* 1.2 Hz) ppm, respectively. The evidence for the localization of the sugar moieties on the aglycone was provided by the ^{13}C NMR of the compound in comparison with kaempferol, which showed the shielding of both C-3 (136.6) and C-7 (163.6), and deshielding for C-4 (179.9), C-2 (159.9), C-8 (95.7) and C-6 (100.6), thus indicating glycosylation sites were at C-3 and C-7. Support for this was shown by the HMBC correlations between the rhamnose anomeric proton at 5.40 with carbon at δ_C 136.6 (C-3) and the another rhamnose anomeric proton at 5.56 with δ_C 163.6 (C-7). The compound was identified as ***kaempferol-3,7-di-O-α-L-rhamnoside*** (*kaempferol-3,7-di-O-rhamnoside)* (**1**).

The ^{1}H NMR spectral data of the aglycone of **2** (see Experimental) indicated an identical pattern to **1**. A comparison of the ^{1}H and ^{13}C NMR spectra of **2** with that of **1**, showed that the signals for H-2, H-3', H-5', H-6', H-6 and H-8, and C-2, C-10, C-1' and C-6' were similar. Thus, the aglycone of **2** is also kaempferol. The ^{1}H NMR spectrum of **2** contained two anomeric proton signals at δ 5.57 (br s), 5.32 (d, *J* 7.2 Hz) attributable to C-3,7-disubstitution of kaempferol. One of these sugars was rhamnose (δ 99.9) suggested by the distinct anomeric proton at 5.57 (br s) and methyl protons at δ 1.25 ppm. The second sugar was identified as *β*-D-glucose by the distinct anomeric proton at *δ* 5.32 (d, *J* 7.2 Hz) correlating with the carbon signal at δ 103.7. The compound was identified as ***kaempferol-3-O-β-glucopyranoside-7-O-α-rhamnoside*** (*kaempferol 3-O-glucosyl-7-O-rhamnoside)* (**2**).

^{1}H and ^{13}C NMR spectral data indicated that **3** is fundamentally a cognate of **2** except for the presence of an additional sugar unit as evidenced by an extra anomeric proton at δ 4.76 and a corresponding anomeric carbon at δ 104.8 in the ^{1}H and ^{13}C NMR, respectively. In the ^{13}C NMR spectrum, the inner glucose was shown to be linked to another terminal one through 1→2 bond on the basis of glucosyl C-2''' downfield shift of ~ + 6.9 ppm at δ 82.7, indicating that the bios is *β*-glucosyl-(1→2)-*β*-glucoside. The HMBC spectrum confirmed the foregoing evidence and correlation peak between the anomeric proton at δ 4.76 (H-1'''') and δ_C 82.7 (C-2''') was observed. This was further supported by the near identity of the relevant ^{1}H, ^{13}C NMR and HMBC data that were in good agreement with the data reported for the *β*-D-glucosyl (1→2)-β-glucoside (**3**) (Markham, R. K.; Geiger, H.; Jagger, H. *Phytochemistry* **1992**, *31*, 1009, Chao, L. R.; Seguin, E.; Tilleguin, F.; Koch, M. *J. Nat. Prod*. **1990**, *53*, 1337, Li, Y. S.; Liu, Y. L. *Phytochemistry* **1990**, *29*, 3311). Thus the compound **3** was identified as ***kaempferol 3-O-β-D-glucopyranosyl-(1→2)-β-D-glucopyranosyl-7-O-α-rhamnopyranoside*** ***(****kaempferol 3-O-glucosyl-(1→2) -glucoside-7-O-rhamnoside)****.***

The differences in the ^{1}H- and ^{13}C-NMR spectra of **3** and **4** suggested that they differ only in the sugar sequence. The ^{1}H- and ^{13}C-NMR chemical shifts, together with 1D-sel TOCSY, COSY, HSQC, HMBC, and H2BC data, confirmed the identity of the sugars as Glc, Gal, and Rha. The ^{1}H- and ^{13}C-NMR data indicated *β*-anomers for Glc and Gal, and *α*-anomer for Rha. The positions of attachment of the sugars moieties to the aglycone were deduced by the ^{1}H,^{13}C long-range correlations observed for anomeric proton of Rha at δ 5.57 (H-1'') and δ_C 163.5 (C-7) and for anomeric proton of Gal at δ 5.39 (H-1''') and δ_C 135.2 (C-3). The C(2''') resonance of the Gal moiety was shifted downfield due to glycosylation and provided the site of attachment of Glc to Gal, which was confirmed by the HMBC correlation from H-1''' of Glc to C-2'''' of Gal. Thus, the structure of compound **4** was established as ***kaempferol 3-O-β-D-glucopyranosyl-(1→2)-β-D-galactopyranosyl-7-O-α-rhamnopyranoside*** ***(****Kaempferol 3-O-glucosyl-(1→2)--galactoside-7-O-rhamnoside)****.***

Kaempferol-3,7-di-O-α-rhamnoside (1)

δ_{1H} (600 MHz, methanol-d_4): 7.80 (2H, d, *J* 8.4 Hz, H-2',6'), 6.94 (2H, d, *J* 8.4 Hz, H-3',5'), 6.74 (1H, d, *J* 2.1 Hz, H-8), 6.47 (1H, d, *J* 2.1 Hz, H-6), 5.56 (1H, d, *J* 1.2 Hz, H-1"), 5.40 (1H, d, *J* 1.8 Hz, H-1'''), 4.22 (1H, dd, *J* 3.0, 1.2 Hz, H-2'''), 4.02 (1H, dd, *J* 3.6, 1.8 Hz, H-2"), 3.83 (1H, dd, *J* 9.0, 3.0 Hz, H-3"), 3.71 (1H, dd, *J* 9.0, 3.0 Hz, H-3'''), 3.59 (1H, t, *J* 9.6 Hz, H-5"), 3.48 (1H, t, *J* 9.6 Hz, H-4"), 3.34 (2H, m, H-4''',5'''), 1.26 (3H, d, *J* 6.0 Hz, H-6"), 0.93 (3 H, d, *J* 5.4 Hz, H-6''').

δ_{13C} (150 MHz, methanol-d_4): 179.9 (C-4), 163.6 (C-7), 163.1 (C-5), 161.8 (C-4'), 159.9 (C-2), 158.2 (C-9), 136.6 (C-3), 132.1 (C-2',6'), 122.5 (C-1'), 116.1 (C-3',5'), 107.6 (C-10), 103.6 (C-1'''), 100.6 (C-6), 99.9 (C-1"), 95.7 (C-8), 73.5 (C-4"), 73.2 (C-4'''), 72.2 (C-5'''), 72.1 (C-3'''), 72.1 (C-3"), 72.0 (C-2'''), 71.8 (C-2"), 71.3 (C-5"), 18.1 (C-6"), 17.7 (C-6''').

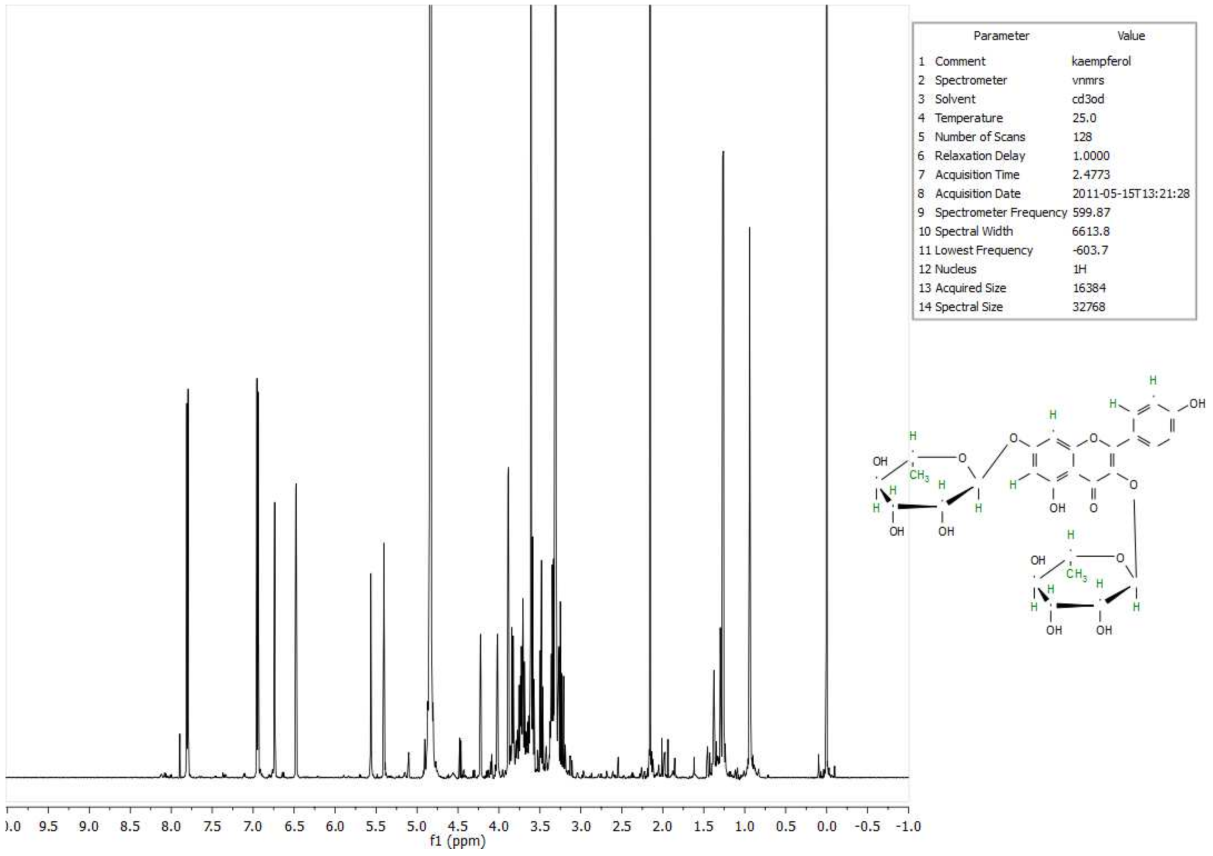

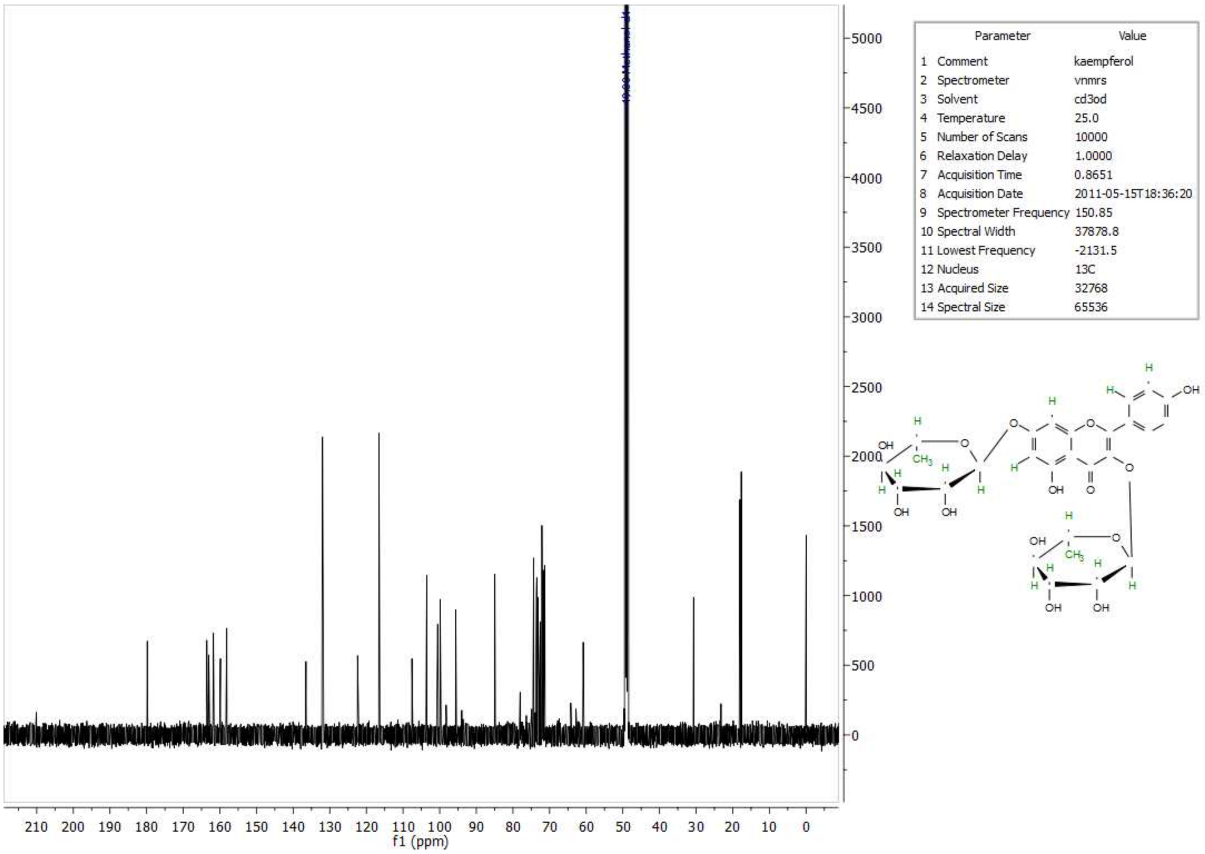

Kaempferol 3-O-β-glucopyranoside-7-O-α-rhamnopyranoside (2)

δ_{1H} (600 MHz, methanol-d_4): 8.08 (2H, d, *J* 8.6 Hz, H-2',6'), 6.90 (2H, d, *J* 8.7 Hz, H-3',5'), 6.77 (1H, d, *J* 2.1 Hz, H-8), 6.47 (1H, d, *J* 2.1 Hz, H-6), 5.57 (1H, s, H-1''), 5.32 (1H, d, *J* 7.2 Hz, H-1'''), 4.03 (1H, s, H-2''), 3.83 (1H, dd, *J* 9.4, 3.3 Hz, H-3''), 3.70 (1H, dd, *J* 11.9, 2.3 Hz, H-6'''a), 3.63–3.56 (1H, m, H-5''), 3.52 (1H, dd, *J* 11.9, 5.6 Hz, H-6'''b), 3.48 (1 H, t, *J* 9.4 Hz, H-4''), 3.46–3.40 (2H, m, H-2''',3'''), 3.30 (1H, m, H-4'''), 3.21 (1H, ddd, *J* 11.9, 5.6, 2.4 Hz, H-5'''), 1.25 (3H, d, *J* 6.2 Hz, H-6'').

δ_{13C} (150 MHz, methanol-d_4): 179.7 (C-4), 163.6 (C-7), 162.9 (C-5), 161.7 (C-4'), 159.6 (C-2), 158.1 (C-9), 135.6 (C-3), 132.4 (C-2',6'), 122.7 (C-1'), 116.1 (C-3',5'), 107.5 (C-10), 103.7 (C-1'''), 100.6 (C-6), 99.9 (C-1''), 95.6 (C-8), 78.5 (C-5'''), 78.0 (C-3'''), 75.8 (C-2'''), 73.6 (C-4''), 72.1 (C-3''), 71.7 (C-2''), 71.4 (C-4'''), 71.3 (C-5''), 62.6 (C-6'''), 18.1 (C-6'').

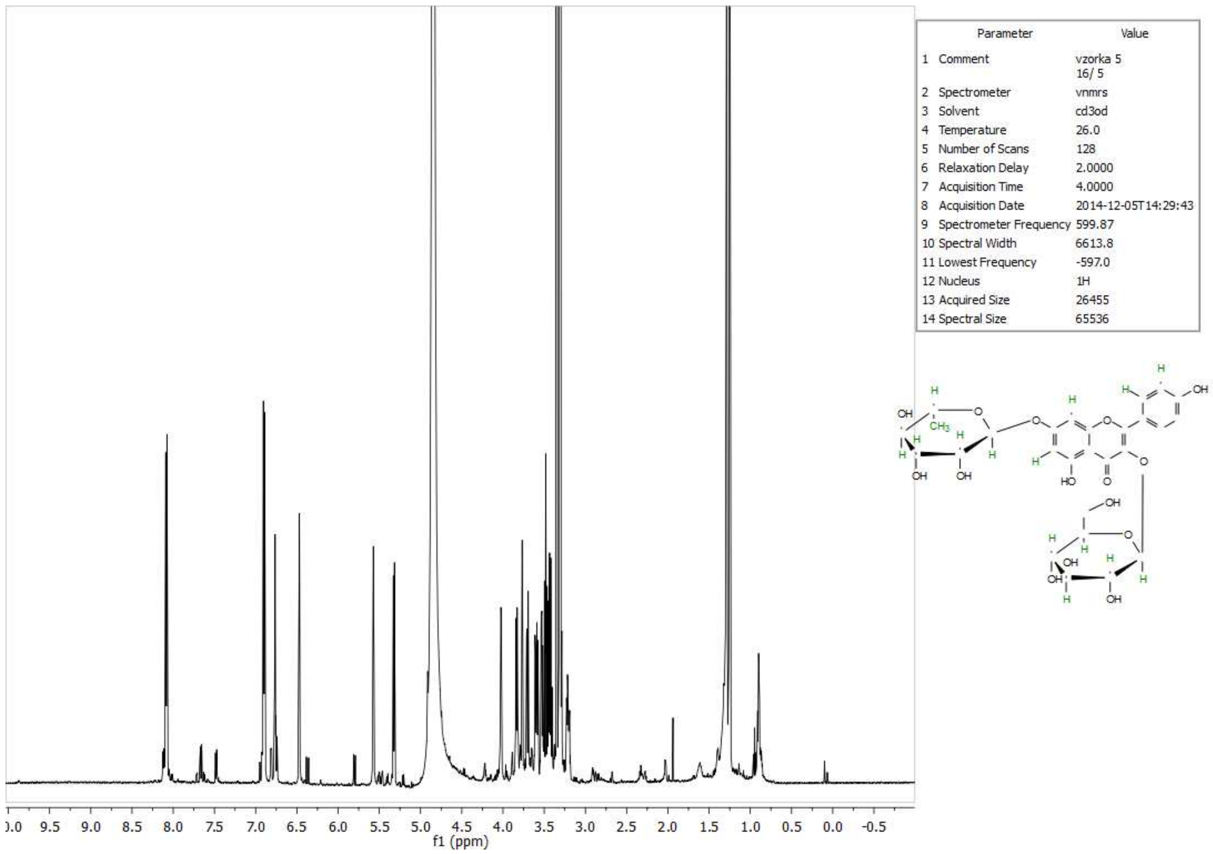

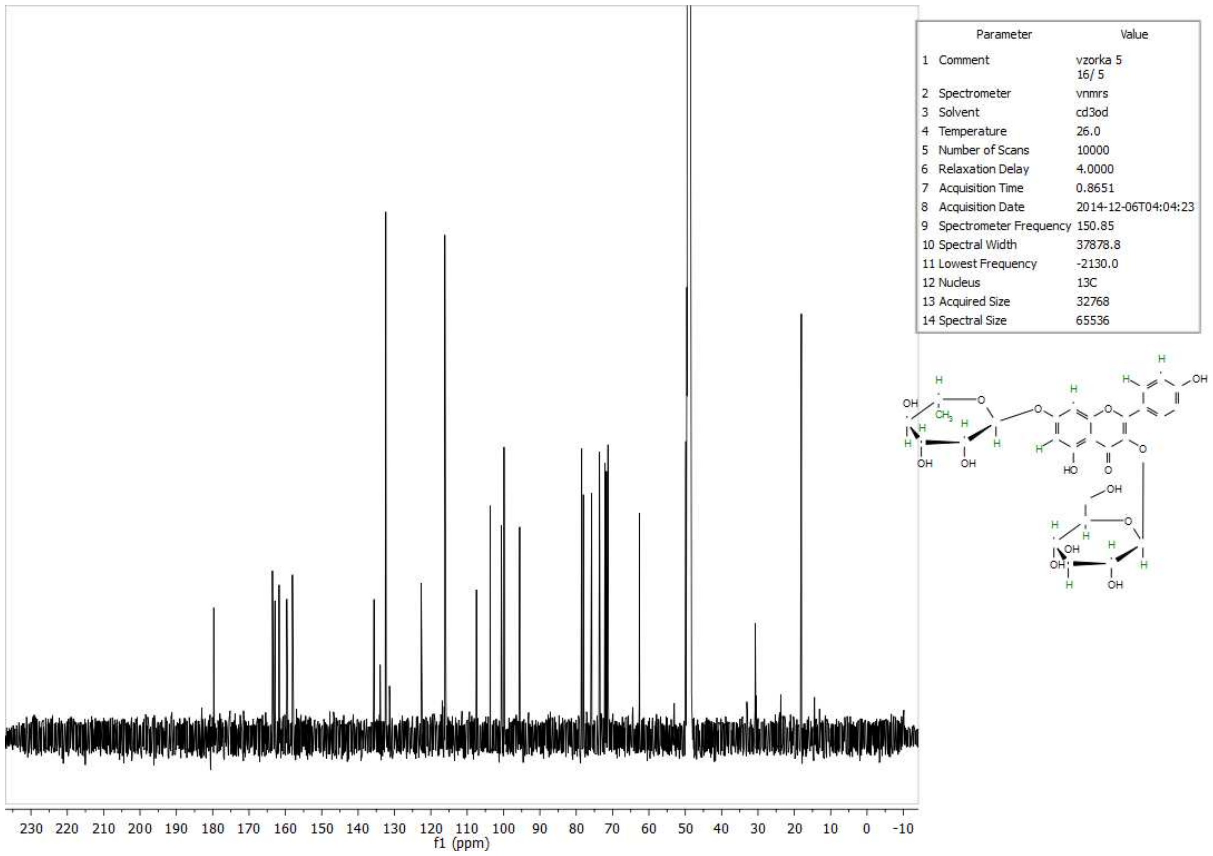

Kaempferol 3-O-β-glucopyranosyl-(1→2)-β-glucopyranoside-7-O-α-rhamnopyranoside (3)

δ_{1H} (600 MHz, methanol-d_4): 8.07 (2H, d, *J* 8.6 Hz, H-2',6'), 6.91 (2H, d, *J* 8.7 Hz, H-3',5'), 6.76 (1H, s, H-8), 6.46 (1H, s, H-6), 5.57 (1H, s, H-1"), 5.48 (1H, d, *J* 7.6 Hz, H-1"'), 4.76 (1H, d, *J* 7.5 Hz, H-1""), 4.03 (1H, s, H-2"), 3.83 (1H, dd, *J* 9.6, 3.6 Hz, H-3"), 3.79 (1H, dd, *J* 12.0, 2.5 Hz, H-6""a), 3.75 (1H, dd, *J* 7.6 Hz, 2"'), 3.69 (1H, dd, *J* 11.9, 2.5 Hz, H-6""b), 3.60 (2H, m, H-5", H-3"'), 3.48 (2 H, m, H-4", H-6"'b), 3.38 (4H, m, H-4"', H-2"", H-3"", H-4""), 3.30 (1 H, m, H-5""), 3.21 (1H, ddd, J 10.2, 5.7, 2.3 Hz, H-5"'), 1.25 (3H, d, *J* 6.2 Hz, H-6").

δ_{13C} (150 MHz, methanol-d_4): 179.8 (C-4), 163.5 (C-7), 162.9 (C-5), 161.7 (C-4'), 159.4 (C-2), 158.0 (C-9), 135.2 (C-3), 132.4 (C-2',6'), 122.6 (C-1'), 116.3 (C-3',5'), 107.5 (C-10), 104.8 (C-1""), 102.0 (C-6), 100.9 (C-1"'), 99.8 (C-1"), 95.5 (C-8), 82.7 (C-2"'), 78.3 (C-5"'), 78.2 (C-5""), 77.9 (C-3"', C-3""), 75.6 (C-2""), 73.6 (C-4"), 72.1 (C3"), 71.7 (C-2"), 71.3 (C-4""), 71.25 (C-5"), 71.2 (C-4"'), 62.6 (C-6""), 62.5 (C-6"'), 18.1 (C-6").

Kaempferol 3-O-β-glucopyranosyl-(1→2)-β-galactopyranoside-7-O-α-rhamnopyranoside (4)

δ_{1H} (600 MHz, methanol-d_4): 8.12 (2H, d, *J* 8.6 Hz, H-2',6'), 6.92 (2H, d, *J* 8.7 Hz, H-3',5'), 6.76 (1H, s, H-8), 6.46 (1H, s, H-6), 5.57 (1H, s, H-1"), 5.39 (1H, d, *J* 7.7 Hz, H-1"'), 4.77 (1H, d, *J* 7.6 Hz, H-1""), 4.06 (1H, dd, *J* 9.5, 7.6 Hz, H-2"'), 4.03 (1H, s, H-2"), 3.85 (1H, d, *J* 3.8 Hz, H-4"'), 3.83 (1H, dd, *J* 9.6, 3.6 Hz, H-3"), 3.79 (1H, dd, *J* 12.0, 2.5 Hz, H-6""a), 3.73 (1H, dd, *J* 9.5, 3.0 Hz, 3"'), 3.69 (1H, dd, *J* 11.9, 2.5 Hz, H-6""b), 3.60 (2H, m, H-5", H-6"'a), 3.54 (1H, dd, *J* 11.4, 6.3 Hz, H-6"'b), 3.49 (1 H, m, H-4") 3.45 (1H, dd, *J* 6.0, 4.8 Hz, H-5"'), 3.38 (3H, m, H-2"", H-3"", H-4""), 3.30 (1 H, m, H-5""), 1.25 (3H, d, *J* 6.2 Hz, H-6").

δ_{13C} (150 MHz, methanol-d_4): 180.0 (C-4), 163.5 (C-7), 162.9 (C-5), 161.7 (C-4'), 159.4 (C-2), 158.0 (C-9), 135.2 (C-3), 132.6 (C-2',6'), 122.5 (C-1'), 116.3 (C-3',5'), 107.4 (C-10), 104.9 (C-1""), 102.0 (C-6), 101.4 (C-1"'), 99.8 (C-1"), 95.5 (C-8), 80.5 (C-2"'), 78.2 (C-5""), 77.9 (C-3""), 77.0 (C-5"'), 75.6 (C-2""), 74.9 (C-3"'), 73.6 (C-4"), 72.1 (C3"), 71.7 (C-2"), 71.3 (C-4""), 71.25 (C-5"), 70.2 (C-4"'), 62.6 (C-6""), 62.0 (C-6"'), 18.1 (C-6").

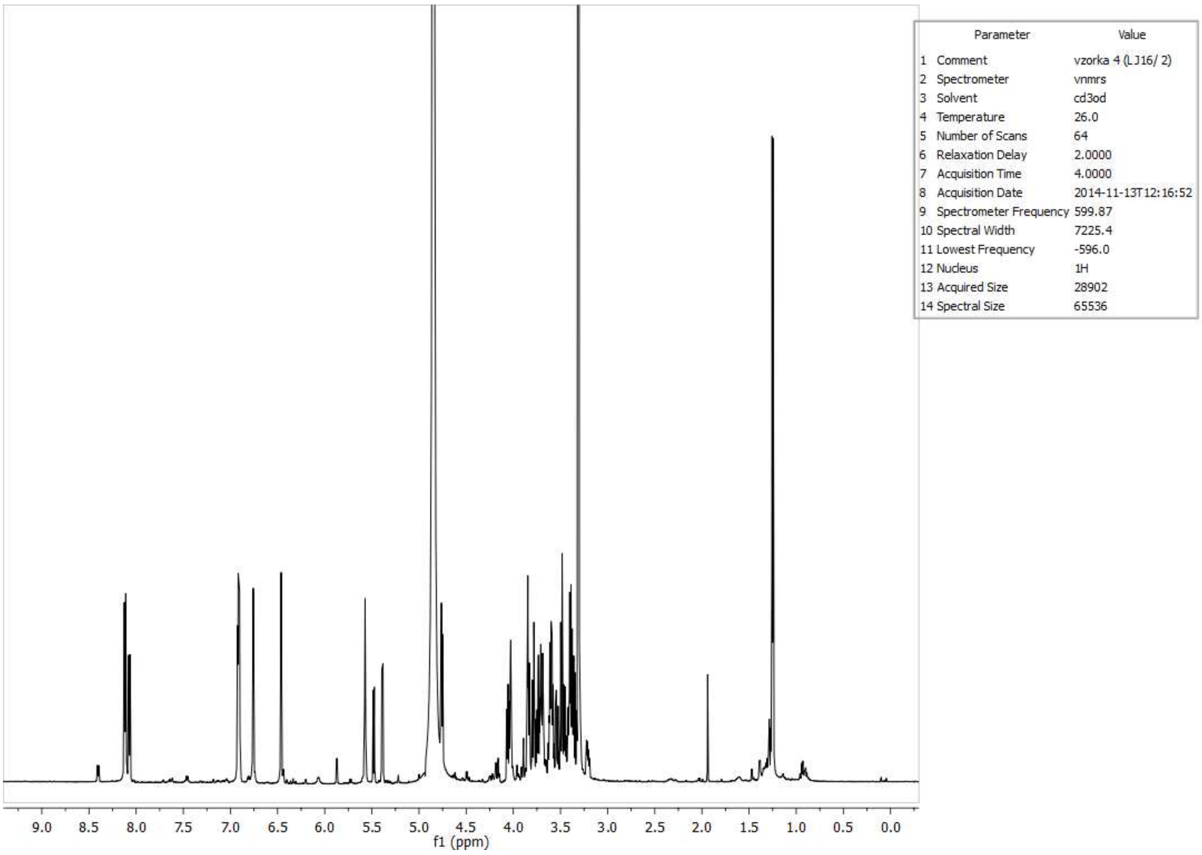

	Parameter	Value
1	Comment	vzorka 4 (LJ16/ 2)
2	Spectrometer	vnmrs
3	Solvent	cd3od
4	Temperature	26.0
5	Number of Scans	64
6	Relaxation Delay	2.0000
7	Acquisition Time	4.0000
8	Acquisition Date	2014-11-13T12:16:52
9	Spectrometer Frequency	599.87
10	Spectral Width	7225.4
11	Lowest Frequency	-596.0
12	Nucleus	1H
13	Acquired Size	28902
14	Spectral Size	65536

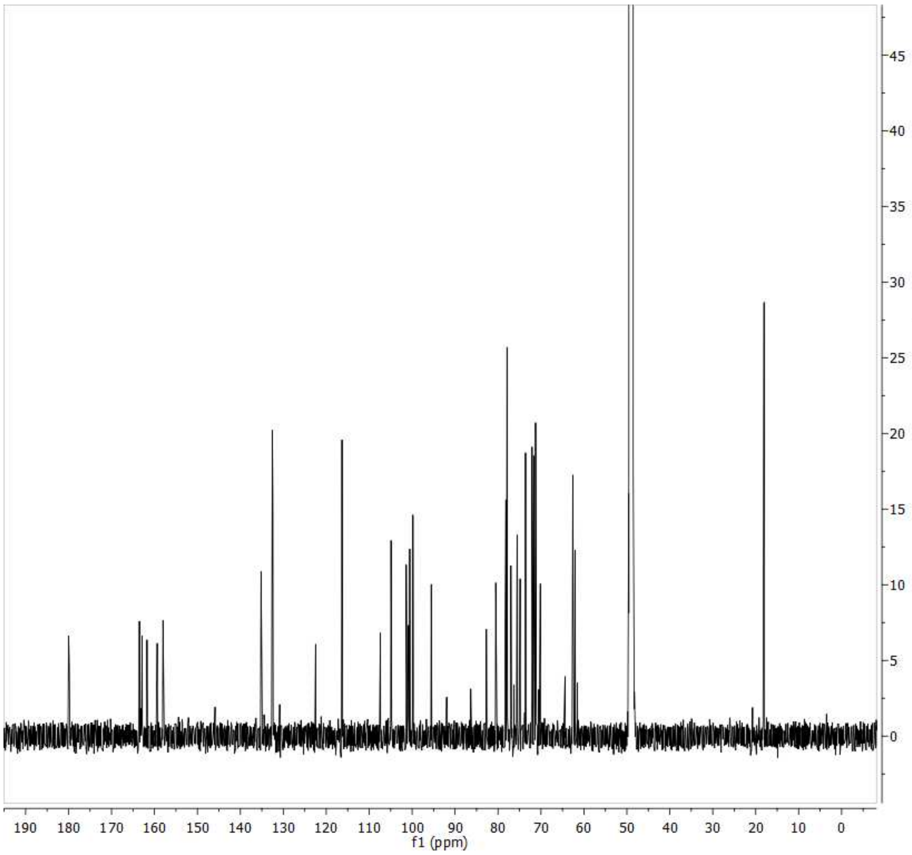

	Parameter	Value
1	Comment	vzorka 4 (LJ16/ 2)
2	Spectrometer	vnmrs
3	Solvent	cd3od
4	Temperature	26.0
5	Number of Scans	5000
6	Relaxation Delay	4.0000
7	Acquisition Time	0.8651
8	Acquisition Date	2014-11-13T01:01:33
9	Spectrometer Frequency	150.85
10	Spectral Width	37878.8
11	Lowest Frequency	-2131.7
12	Nucleus	13C
13	Acquired Size	32768
14	Spectral Size	65536
