# Supplementary material for: Modulation of phenolic metabolism under stress conditions in a Lotus japonicus mutant lacking plastidic glutamine synthetase
Source: Front Plant Sci. 2015 Sep 25;6:760. doi: 10.3389/fpls.2015.00760 (PMC4585329; doi:10.3389/fpls.2015.00760)
Supplement: Supplemental Table S3 — Top 20 genes that were induced bydrought or active photorespiration in the Ljgln2-2 mutant. The highlighted genes are those that were in common among the the list of 20 most induced genes that were observed in the drought stress or active PR treatments. [file Table3.DOCX]

| **Drought** |  |  |  |
| --- | --- | --- | --- |
| **Probeset** | **Locus** | **Fold change** | **Gene product** |
| chr1.CM0012.67_at | chr1.CM0012.2250.r2.d | 294.6 | Thaumatin-like protein |
| chr5.CM0519.59.1_at | n.a. | 39.0 | Retrotransposon protein |
| Ljwgs_010070.2_s_at | chr1.CM2121.240.r2.m | 35.4 | Protein phosphatase 2C |
| Ljwgs_084631.1_s_at | n.a. | 29.9 | Retrotransposon protein |
| chr3.CM0111.37_at | chr3.CM0111.250.r2.m | 29.6 | Mitochondrial chaperone BCS1 |
| chr4.CM0244.91_at | chr4.CM0244.1010.r2.m | 25.1 | Xyloglucan endotransglucosylase |
| Ljwgs_052805.1_at | chr3.CM0786.370.r2.a | 21.6 | Heat shock protein |
| Ljwgs_030984.1_at | LjSGA_030984.1 | 21.0 | Mitochondrial phosphate translocator |
| chr3.CM0091.34_at | chr3.CM0091.590.r2.m | 17.2 | Hypothetical protein |
| chr1.CM0980.20_at | chr1.CM0029.190.r2.m | 16.7 | Hypothetical protein |
| Ljwgs_027307.1_at | LjSGA_027307.1 | 16.4 | Hypothetical peroxygenase |
| chr4.CM0126.95_at | chr4.CM0126.600.r2.m | 15.7 | Stay-green protein |
| chr5.CM0200.109_at | chr5.CM0200.2620.r2.m | 15.2 | Zinc finger protein |
| Ljwgs_016353.2.1_at | LjSGA_016353.2.1 | 12.8 | Hypothetical protein |
| chr2.CM0074.3_at | chr2.CM0074.440.r2.m | 12.6 | Aromatic ring-opening dioxygenase |
| chr5.CM0096.7_s_at | chr5.CM0096.950.r2.d | 12.4 | Hypothetical protein |
| chr5.CM0200.56_at | chr5.CM0200.3220.r2.m | 12.4 | Flavonoid UDP-glycosyltransferase |
| Ljwgs_019489.1_at | LjSGA_019489.1 | 11.6 | Aspartic protease |
| chr2.CM0250.2_at | chr2.CM0250.30.r2.m | 11.3 | Isoflavone 2'-hydroxylase |
| TM1670.18_at | chr2.CM0826.330.r2.m | 11.2 | LysM type receptor kinase (LYS13) |
|  |  |  |  |
| **Active PR** |  |  |  |
| **Probeset** | **Locus** | **Fold change** | **Gene product** |
| chr2.CM0250.2_at | chr2.CM0250.30.r2.m | 20.2 | Isoflavone 2'-hydroxylase |
| TC18455_at | n.a. | 16.6 | Unknown |
| TM1670.18_at | chr2.CM0826.330.r2.m | 16.2 | LysM type receptor kinase (LYS13) |
| chr5.CM0071.54.1_at | chr5.CM0071.380.r2.d | 14.6 | LjMYB15 |
| chr5.CM0519.59.1_at | n.a. | 14.2 | Retrotransposon protein |
| gi45635475_at | n.a. | 12.6 | Hypothetical protein |
| gi45348662_at | n.a. | 11.5 | Hypothetical protein |
| chr5.CM0200.121_at | chr5.CM0200.2510.r2.m | 11.3 | Hypothetical protein |
| chr3.CM0111.37_at | chr3.CM0111.250.r2.m | 11.3 | Mitochondrial chaperone BCS1 |
| chr3.CM0634.22_at | chr3.CM0634.190.r2.m | 10.4 | Acid phosphatase 1 |
| chr1.CM0980.20_at | chr1.CM0029.190.r2.m | 9.9 | Hypothetical protein |
| Ljwgs_030984.1_at | LjSGA_030984.1 | 9.9 | Mitochondrial phosphate translocator |
| chr4.CM0182.42_at | chr4.CM0182.320.r2.m | 9.7 | Alternative oxidase |
| Ljwgs_024517.1_at | LjSGA_024517.1 | 9.6 | Xyloglucan endotransglucosylase |
| Ljwgs_147747.1_at | n.a. | 9.4 | Glutathione S-transferase |
| chr1.CM0591.49_at | chr1.CM0591.620.r2.d | 9.3 | Hypothetical protein |
| chr5.CM0569.37.1_at | chr3.CM0416.1150.r2.a | 9.0 | Receptor-like kinase |
| Ljwgs_019489.1_at | LjSGA_019489.1 | 8.5 | Aspartic protease |
| chr1.TM0811.11_at | chr1.CM0122.2730.r2.d | 8.4 | Putative LEA protein |
| Ljwgs_087148.2_s_at | chr2.CM0803.300.r2.m | 8.2 | Alternative oxidase |

**Supplemental Table S3.** The 20 most highly induced gene probesets under either drought or active PR treatments are listed. The gene probesets and corresponding gene products that were in common among the 20 most highly induced in drought or active PR are highlighted.n.a.: the sequence recognized by the probeset was not available in the current release of the *L. japonicus* genome.
