# Supplementary material for: Modulation of phenolic metabolism under stress conditions in a Lotus japonicus mutant lacking plastidic glutamine synthetase
Source: Front Plant Sci. 2015 Sep 25;6:760. doi: 10.3389/fpls.2015.00760 (PMC4585329; doi:10.3389/fpls.2015.00760)
Supplement: Supplemental Table S4 — List of the available gene sequences for the biosynthesis of phenolic compounds found in the current release of the L. japonicus genome. Data mining was carried out in the 2.5 release of L. japonicus genome at the Kazusa institute database (http://www.kazusa.or.jp/lotus/). The gene names for the members of each gene family were defined in this work or, when available, taken from the bibliography. [file Table4.DOCX]

Supplemental Table S4.

| **Gene product / Pathway** | **Gene name** | **Locus (Kazusa 2.5)** |
| --- | --- | --- |
| ***General phenylpropanoid pathway*** |  |  |
| **Phenylalanine ammonia lyase (PAL)^a^** |  |  |
|  | *LjPAL1* | chr1.CM0012.1040.r2.m |
|  | *LjPAL2* | chr5.LjT17D03.50.r2.d |
|  | *LjPAL3* | chr1.CM0012.1200.r2.m |
|  | *LjPAL4* | chr1.CM0012.1070.r2.m |
|  | *LjPAL5* | chr1.CM0012.1090.r2.m |
|  | *LjPAL6* | chr2.CM0191.870.r2.m |
|  | *LjPAL7* | chr3.CM0574.80.r2.d |
|  | *LjPAL8* | chr1.CM0012.1210.r2.m |
|  | *LjPAL9* | chr1.CM0012.1060.r2.m |
|  | *LjPAL10* | chr5.CM1729.190.r2.m |
| **Cinnamate 4-Hydroxylase (C4H)** |  |  |
|  | *LjC4H1* | LjSGA_003295.2 |
|  | *LjC4H2* | chr5.LjT24B10.140.r2.m |
|  |  |  |
| **4-Coumarate:CoA ligase (4CL)** |  |  |
|  | *Lj4CL1* | chr2.CM0018.1290.r2.m |
|  | *Lj4CL2* | chr3.CM0127.410.r2.m |
|  | *Lj4CL3* | chr3.LjB21L17.130.r2.a |
|  | *Lj4CL4* | chr4.CM0061.190.r2.m |
|  | *Lj4CL5* | chr6.CM0057.360.r2.m |
| ***Biosynthesis of flavonoids*** |  |  |
| **Chalcone synthase (CHS)^b^** |  |  |
|  | *LjCHS1* | chr1.CM0104.1140.r2.m |
|  | *LjCHS2* | chr1.CM0284.240.r2.m |
|  | *LjCHS3* | chr1.CM0284.250.r2.m |
|  |  | chr2.CM0018.760.r2.m |
|  |  | chr2.CM0018.730.r2.m |
|  |  | chr2.CM0018.1150.r2.m |
|  |  | chr3.CM0590.840.r2.m |
|  | *LjCHS12* | chr3.CM0590.770.r2.d |
|  |  | chr4.CM0046.110.r2.m |
|  |  | chr4.CM0044.260.r2.d |
|  |  | chr6.CM0057.460.r2.m |
| **Chalcone isomerase (CHI)^b^** |  |  |
|  | *LjCHI1* | chr5.CM0180.670.r2.m |
|  | *LjCHI2* | chr5.CM0180.690.r2.m |
|  | *LjCHI3* | chr5.CM0180.660.r2.m |
|  | *LjCHI4* | chr5.CM0180.680.r2.m |
| **Flavanone 3β-Hydroxylase (F3H)** |  |  |
|  | *LjF3H1* | chr4.CM0119.240.r2.m |
|  | *LjF3H2* | chr3.LjB14O06.120.r2.a |
| **Flavonol synthase (FLS)** |  |  |
|  | *LjFLS1* | chr1.LjT35H06.80.r2.m |
|  | *LjFLS2* | chr1.LjB17A22.120.r2.m |
|  | *LjFLS3* | chr1.LjT46A21.140.r2.a |
| ***Formation of anthocyanins*** |  |  |
| **Dihydroflavonol reductase (DFR)^c^** |  |  |
|  | *LjDFR1* | chr5.CM0077.120.r2.m |
|  | *LjDFR2* | chr5.CM0077.210.r2.m |
|  | *LjDFR3* | chr5.CM0077.140.r2.m |
|  | *LjDFR4* | chr5.CM0077.110.r2.m |
|  | *LjDFR5* | chr5.CM0077.150.r2.m |
| **Anthocyanidin synthase (ANS)** |  |  |
|  | *LjANS1* | chr2.CM0021.2820.r2.m |
|  | *LjANS2* | chr1.LjB18K24.110.r2.a |
| ***Formation of proanthocyanidins*** |  |  |
| **Leucoanthocyanidin reductase (LAR)^d^** |  |  |
|  | *LjLAR1* | chr2.CM0124.20.r2.m |
|  | *LjLAR2* | LjSGA_076819.1 |
| **Anthocyanidin reductase (ANR)** |  |  |
|  | *LjANR* | chr4.CM1616.680.r2.m |
| ***Formation of isoflavonoids and derivatives*** |  |  |
| **CHS and CHI: see above** |  |  |
|  |  |  |
| **Polyketide reductase (PKR or CHR)^b^** |  |  |
|  | *LjPKR1* | chr2.CM0191.470.r2.m |
|  |  | chr2.CM0191.480.r2.m |
|  | *LjPKR3 (Pseudogene)* | chr2.CM0191.490.r2.m |
|  |  | chr2.CM0191.560.r2.m |
|  |  | chr5.CM0345.940.r2.m |
|  |  | chr6.CM0037.960.r2.m |
| **Isoflavone synthase (IFS or 2HIS)^b^** |  |  |
|  | *LjIFS1* | chr4.CM0432.2900.r2 |
|  | *LjIFS2* | chr4.CM0432.3190.r2.m |
|  | *LjIFS3*  *(Pseudogene)* | chr4.CM0432.3150.r2.m |
| **2,4,7’-Hydroxyisoflavanone 4’-O-methyltransferase (HI4’OMT)** |  |  |
|  | *LjHI4’OMT* | chr4.CM0432.2880.r2.m |
| **Isoflavanone dehydratase (HID or IFD)^b^** |  |  |
|  | *LjHID* | chr5.CM0200.1460.r2.m |
| **Isoflavone 2’-hydroxylase (I2’H)^b^** |  |  |
|  | *LjI2’H1* | chr2.CM0250.70.r2.m |
|  | *LjI2’H2* | chr4.CM0026.1220.r2.a |
| **Isoflavone reductase (IFR)^b^** |  |  |
|  | *LjIFR1* | chr2.CM0249.1380.r2.m |
|  | *LjIFR2* | chr2.CM0249.1390.r2.m |
| **Vestitone reductase (VR)^b^** |  |  |
|  | *LjVR1* | chr1.CM1255.110.r2.m |
|  | *LjVR2* | chr1.CM1255.100.r2.m |
| **Pterocarpan reductase (PTR)^e^** |  |  |
|  | *LjPTR1* | chr3.CM0091.1150.r2.d |
|  | *LjPTR2* | chr3.CM0091.1170.r2.d |
|  | *LjPTR3* | chr3.CM0091.1180.r2.d |
|  | *LjPTR4* | chr1.CM0952.30.r2.a |

^a^Gene names according to Deguchi et al., 2007.

^b^Gene names according to Shimada et al., 2007 (when possible).

^c^Gene names according to Shimada et al., 2005

^d^Gene names according to Takos and Rook, 2014

^e^Gene names according to Akashi et al., 2006.

**REFERENCES**

Akashi, T., Koshimizu, S., Aoki, T. and Ayabe, S.-i. (2006). Identification of cDNAs encoding pterocarpan reductase involved in isoflavan phytoalexin biosynthesis in Lotus japonicus by EST mining. *FEBS Lett.* 580, 5666-5670.

Deguchi, Y., Banba, M., Shimoda, Y., Chechetka, S.A., Suzuri, R., Okusako, Y. et al. (2007). Transcriptome profiling of *Lotus japonicus* roots during arbuscular mycorrhiza development and comparison with that of nodulation. *DNA Res.* 14, 117-133.

Shimada, N., Sasaki, R., Sato, S., Kaneko, T., Tabata, S., Aoki, T. et al. (2005). A comprehensive analysis of six dihydroflavonol 4-reductases encoded by a gene cluster of the *Lotus japonicus* genome. *J. Exp. Bot.* 419, 2573-2585.

Shimada, N., Sato, S., Akashi, T., Nakamura, Y., Tabata, S., Ayabe, S.-i. and Aoki, T. (2007). Genome-wide analyses of the structural gene families involved in the Legume-specific 5-Deoxyisoflavonoid Biosynthesis of *Lotus japonicus*. *DNA Res.* 14, 25-36.

Takos, A.M. and Rook, F. (2014). “Plant-specialized metabolism and its genomic organization in biosynthetic gene clusters in Lotus japonicus” in *The Lotus japonicus genome*, ed. S. Tabata and J. Stougaard (Heidelberg, Germany, Springer-Verlag), 149-162.
