# Supplementary material for: Modulation of phenolic metabolism under stress conditions in a Lotus japonicus mutant lacking plastidic glutamine synthetase
Source: Front Plant Sci. 2015 Sep 25;6:760. doi: 10.3389/fpls.2015.00760 (PMC4585329; doi:10.3389/fpls.2015.00760)
Supplement: Supplemental Table S5 — LC/ESI-MS data of flavonol glycosides identified in 50% methanol extracts from L. japonicus leaves. Correspondence between peak numbers and metabolites as described in the legend of Figure 5. [file Table5.DOCX]

**Supplemental Table S5. LC/ESI-MS data of flavonol glycosides identified in 50% methanol extracts from *L. japonicus* leaves.**

| **Peak No.** | **R_t_**  **(min.)** | **λ_max_**  **(nm)** | **Molecular**  **[M+H]^+^** | **Fragments** | **Compound** |
| --- | --- | --- | --- | --- | --- |
| **1** | 8,4 | 265, 347 | 757 | 595, 433, 287 | Kae 3-O-Glu-Glu 7-O-Rha |
| **2** | 9,0 | 265, 347 | 757 | 595, 433, 287 | Kae 3-O-Glu-Gal 7-O-Rha |
| **3** | 12,2 | 255, 354 | 611 | 449, 303 | Que-6DH, -Hex |
| **4** | 13,2 | 256, 354 | 611 | 449, 303 | Que-6DH, -Hex |
| **5** | 15,8 | 265, 346 | 595 | 433, 287 | Kae 3-O-Gal 7-O-Rha |
| **6** | 17 | 265, 346 | 595 | 433, 287 | Kae 3-O-Glu 7-O-Rha |
| **7** | 17,3 | 255, 349 | 595 | 449, 303 | Que-6DH, -6DH |
| **9** | 21,4 | 264, 343 | 579 | 433, 287 | Kae 3-O-Rha 7-O-Rha |
| **12** | 33,8 | 265, 365 | 433 | 287 | Kae-6DH |
| **13** | 36,9 | 252, 270, 365 | 433 | 287 | flavonol-6DH |

Kae – kaempferol, Que – quercetin, Rha – rhamnose, Glu – glucose, Gal – galactose, Hex – hexose, 6DH – 6-deoxyhexose
